# Metabolic profiling and antimicrobial activity of *Bistorta amplexicaulis* D. don by *in-vitro* implicated through computational studies

**DOI:** 10.3389/fphar.2025.1575727

**Published:** 2025-07-01

**Authors:** Hanan Javid, Umar Yousuf, Roof Ul Qadir, Junaid A. Magray, Bilal A. Wani, Tajamul Islam, Irshad A. Nawchoo, Shabana Gulzar

**Affiliations:** ^1^ Plant Reproductive Biology, Genetic Diversity and Phytochemistry Research Laboratory, Department of Botany, University of Kashmir, Srinagar, India; ^2^ Center of Research for Development (CORD), University of Kashmir, Srinagar, India

**Keywords:** *Bistorta amplexicaulis*, metabolic profiling, antimicrobial activity, computational studies, ADMET analysis

## Abstract

**Ethnopharmacological relevance:**

*Bistorta amplexicaulis* is an important medicinal plant from the Polygonaceae family. It is been utilized traditionally to cure several ailments. However, its essential bioactivities and antimicrobial mechanisms have remained unexplored.

**Aim of the study:**

The present study aimed to investigate the antimicrobial mechanisms of various extracts of *B. amplexicaulis* through the application of standard antimicrobial assays. We investigated by molecular dynamics (MD) simulation studies, the possible targets of one of the compounds found in this plant.

**Materials and methods:**

The qualitative and quantitative analysis of phytochemicals was performed using established methodologies. Additionally, high-resolution liquid chromatography-mass spectrometry (HR/LC-MS) analysis was carried out on the active extracts to identify the secondary metabolites present in various parts of *B. amplexicaulis*. Moreover, using *in vitro* methods, these extracts have been tested for antimicrobial activity against a variety of bacterial (*Bacillus subtilus, Staphylococcus aureus, Proteus vulgaris, Escherichia coli*) and fungal (*Aspergillus flavus, Aspergillus niger, Penicillium notatum*) strains. Besides, molecular modeling of identified compounds was conducted against various crucial microbial drug target proteins.

**Results:**

Metabolic profiling demonstrates that around 22 and 35 bioactive compounds are identified from the belowground and the aboveground parts, respectively, and many of these compounds have therapeutic uses. Further, ethyl acetate extracts from the underground parts showed the widest Inhibition Zone Diameter (IZD) at 18.07 ± 0.38 mm against *B. subtilus* at 1,000 μg/mL, and the smallest IZD was shown by methanolic extracts of aboveground parts against *P. vulgaris* (5.50 ± 0.39 mm). The inhibitory activity of various doses of plant extracts against *A. niger, A. flavus,* and *Penicillium notatum* was also tested. At a concentration of 600 μg/mL, ethyl acetate extracts from the underground parts exhibited the most significant inhibition against *Penicillium notatum*, leading to an 84.56% ± 2.56% reduction in mycelial growth compared to the control. In contrast, the lowest inhibition was observed in methanol extracts from the aboveground parts against *A. flavus*, resulting in a 26.18% ± 2.58% inhibition in mycelial growth. In addition, molecular docking and MD simulation studies on the compounds revealed significant binding affinity, supporting the observed *in-vitro* antimicrobial activity.

**Conclusion:**

Overall, this study offers an extensive understanding of the chemical composition of *B. amplexicaulis* extracts and their antimicrobial potential. Furthermore, Computational studies have provided deep insights into how plant secondary metabolites interact with microbial drug target proteins, leading to more targeted and effective antimicrobial therapies.

## Introduction

In the annals of human civilization and across diverse geographical regions, the plant kingdom has been a rich source of medicinal remedies. Alkaloids, flavonoids, tannins, and phenolic compounds are among the most prominent bioactive constituents responsible for the therapeutic properties of these plants (Thirumurugan et al., 2010). The concentrations of these compounds differ among plant species, contributing to the distinctive medicinal attributes of each plant. Novel therapeutic agents may be discovered as a result of the growing interest in traditional ethnomedicine. Phytochemicals having notable activity against human pathogenic bacteria are found in many plant species, many of which have been studied pharmacologically and therapeutically (Khyade et al., 2009; Bhat et al., 2022a; Mir et al., 2022a).

Antimicrobial drugs are essential for reducing the prevalence of infectious disorders worldwide (Bhatia and Narain, 2010; Sheikh et al., 2022). On the other hand, the spread and appearance of pathogenic microorganisms that are resistant to drugs (MDR) represents a serious risk to public health, leading to a scarcity of effective antimicrobial agents for combating bacterial infections (Boucher et al., 2009; Giamarellou, 2010; Tabasum et al., 2022). The advances in the field of antimicrobial drugs are threatened by the emergence of resistant bacteria and have been shown to cause an estimated >1.2 million deaths per year worldwide (Murray et al., 2022) Therefore, antimicrobial resistance is acknowledged as one of the major global public health threats by the World Health Organization (WHO) and the U.S. Centers for Disease Control and Prevention (CDC) Tängdén et al. (2024). Antibiotic resistance in bacteria can be acquired, adaptive, or intrinsic. Intrinsic resistance is the resistance exhibited as a result of the bacterium’s natural traits (Almutairy, 2024) Acquired resistance is the term used to describe the development of a resistance mechanism in a previously susceptible bacteria through mutation or the acquisition of new genetic material from an external source (horizontal gene transfer) (Christaki et al., 2020) Consequently, the urgent need to discover new antimicrobial agents has spurred a growing interest in traditional medicine, which offers natural, eco-friendly remedies devoid of adverse effects (Bhat et al., 2022b; Sahoo and Manchikanti, 2013). Despite the advantages of synthetic medicines, plant-based natural remedies remain popular choices (Sheikh et al., 2021; Mir et al., 2022b) owing to the diverse phytoconstituents found in medicinal plants that enable the treatment of various human health issues (Anand et al., 2019; Yaseen et al., 2023).

In the realm of drug discovery, computational methods have become indispensable tools for screening drugs made from phytochemicals found in medicinal plants. Computer-aided approaches aid in the prediction of pharmacokinetic, pharmacological, and toxicological properties, guiding pharmaceutical and technological research methodologies (Mayr et al., 2018; Tariq et al., 2021). Molecular docking: an economical and successful approach, that enables the development and testing of pharmaceuticals by predicting drug-receptor interactions and the binding orientation of drug candidates to their target proteins (Ramsundar et al., 2017). This approach allows for a systematic exploration of ligand binding at active sites, facilitating the identification of potential therapeutic agents (Hopkins and Groom, 2002; Unterthiner et al., 2015).

In the specific context of this study, the medicinal plant *Bistorta amplexicaulis (Syn: Polygonum amplexicaule)* was investigated. The plant species belongs to the family Polygonaceae and is native to Asia, mostly found in Afghanistan, Nepal, China, India, and Pakistan (Gairola et al., 2014; Khan et al., 2011) This plant holds medicinal significance, offering remedies for conditions such as infectious diseases (Jabeen et al., 2022; Majid et al., 2020; Maroyi, 2013; Shah et al., 2020), circulatory issues, muscle injuries, stomach pain, fractures, osteoporosis, rheumatism, and oral inflammation (Xiang et al., 2011; Xie et al., 1996). The study aimed to identify the bioactive components in the plant extract using HR-LC/MS analysis and the quantification of phenolics, flavonoids, and tannins to assess the *in-vitro* antimicrobial activity of different parts of *B. amplexicaulis* explore the impact of solvent polarity on the antimicrobial activity of various extracts, including ethyl acetate and methanol, and analyze the most prevalent substances *in silico* against target proteins that are involved in the life cycles of fungus and bacteria.

## Material and methods

### Ethical considerations

The plant material used in this study was collected in compliance with all relevant local and national regulations governing the collection and use of plant resources. Necessary permissions were obtained from the appropriate authorities, and efforts were made to ensure sustainable and ethical sourcing practices. The plants were recognized in Kashmir University Herbarium (KASH) with voucher specimen No. 4319.

### Study area

**FIGURE 1:**
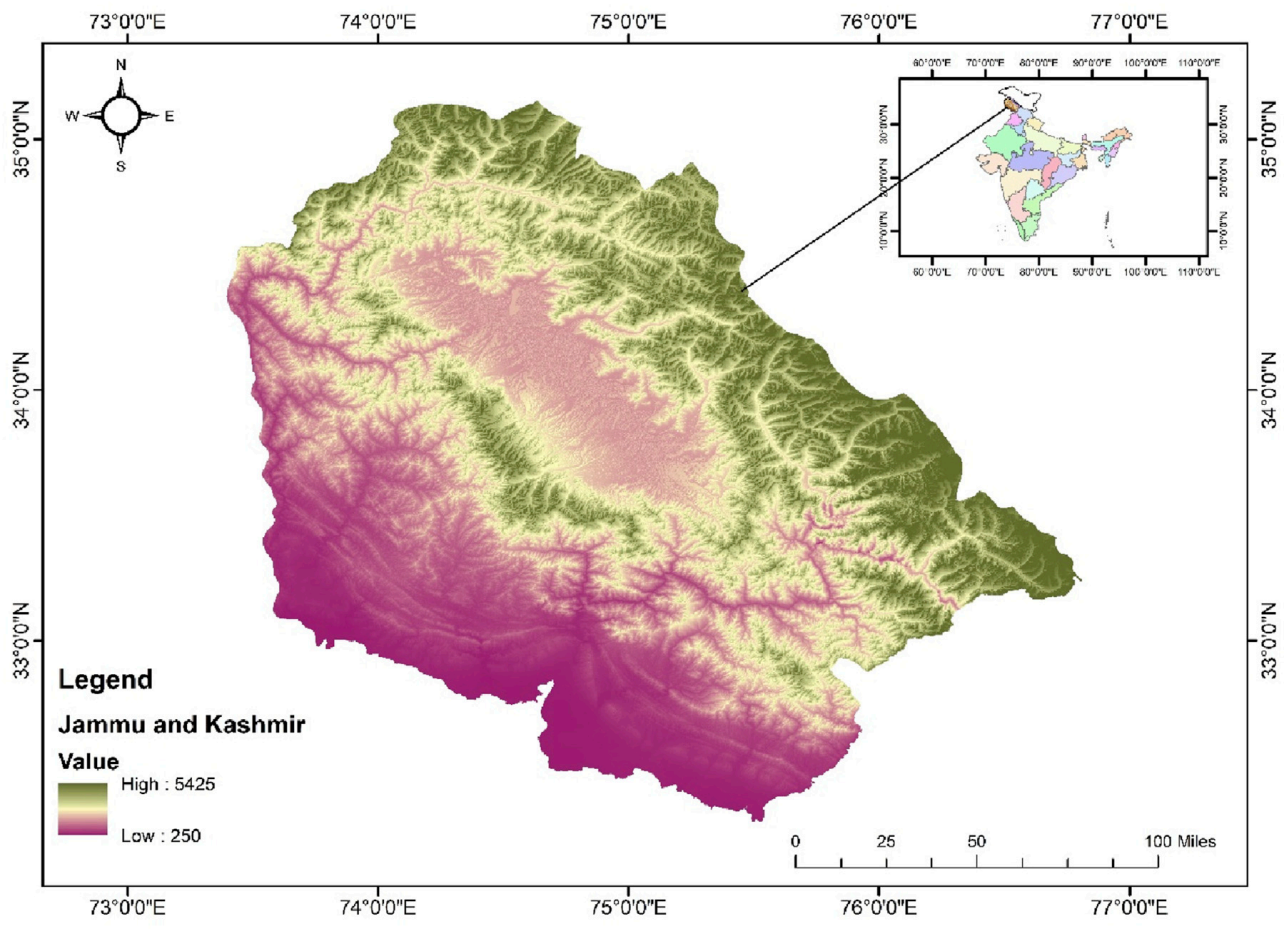
Map of the Study area in Kashmir belt (UT of Jammu and Kashmir) India.

The study was carried out in Kashmir Himalaya, a distinct biosphere unit located in the northern Himalayas, notable for its significant biogeographical position (Figure 1). Encompassing an area of approximately 15,948 km^2^, nearly 64% of this region consists of mountains. Geographically, it lies between 32°20′and 34°50′North latitude and 73°55′and 75°35′East longitude. It is characterized by a deep, elliptical, bowl-shaped valley, bordered to the south and southwest by the Pir Panjal range of the Lesser Himalaya, and to the north and northeast by the Zanskar range of the Greater Himalaya. While the surrounding mountains have an average elevation of from 3,000 to 4,000 m, the valley is located between 1,500 and 1800 m above sea level (asl) (Romshoo et al., 2020). The climate in this area follows a temperate latitudinal pattern, resembling the continental Mediterranean type, characterized by distinct seasonal variations.

### Soxhlet extraction

**FIGURE 2:**
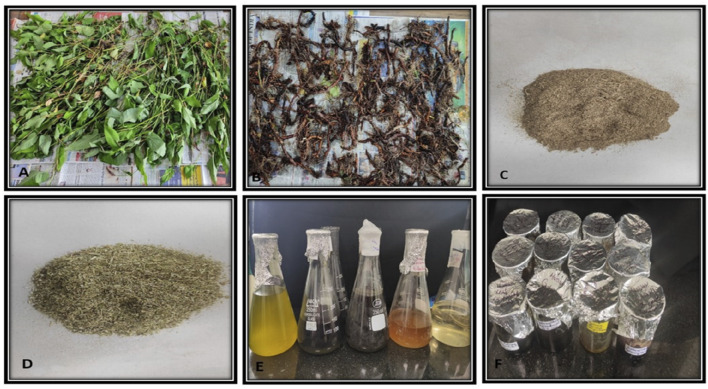
Preparation of plant extracts **(A,B)** Shade drying of plant material **(C,D)** powdered plant material **(E)** solvent after soxhlet extraction **(F)** final extract.

To conduct the phytochemical analysis, fully grown flowering plants (20–30) were randomly selected from designated populations. The plants were then separated into their respective parts: rhizomes, stems, leaves, and flowers. Rhizomes were cleaned meticulously with running tap water, then sterilized using 70% ethanol for 2 min, followed by rinsing with distilled water. The plants were air-dried in the shade for 15–20 days and subsequently ground into a fine powder using an electric grinder. Approximately 50 g of plant material (dried form) from both aboveground (Stem, leaf, and flower) and belowground (Rhizome) parts from all the specified sites underwent hot successive extraction in a Soxhlet apparatus using 500 mL of different solvents (Petroleum ether, Ethyl acetate, Methanol, and Aqueous solutions) (Bimakr et al., 2011). The extracts were passed through Whatman filter paper no. 1, and the filtrates were concentrated using a rotary evaporator. The dried extracts were subsequently stored in sealed glass vials at 4°C until further analysis (Figure 2).

### Qualitative and quantitative analyses of phytochemicals

In this research study, the analysis of phytochemicals present in *B. amplexicaulis* extracts was conducted employing both qualitative and quantitative methods. The qualitative analysis, as per standard procedures given by Harborne, (1998), Parekh and Chanda (2007), Sadasivam and Manickam (1996) was performed to identify the presence of phytochemical compounds. Quantification of total flavonoids was conducted by following the methodology by Oguyemi, 1979 (Oguyemi and Sofowora, 1979). This technique involves the formation of a complex between flavonoids and aluminum, showing maximum absorbance at 415Å. Rutin solution (0.5 mg/mL) in methanol was used as a standard, and the absorbance was measured accordingly (Kumaran and Joel karunakaran, 2006). The total phenolic content in the plant extracts was measured by recording the absorbance at 765 nm, using the standard protocol outlined by Hagerman et al. (2000) (Joint FAO/IAEA Division of Nuclear Techniques in Food and Agriculture, 2000). Tannins were quantified following the methodology given by Saeed et al. (2012).

### Liquid chromatography coupled with high-resolution mass spectrometry

To further investigate the bioactive compounds, present in *B. amplexicaulis*, high-resolution liquid chromatography-mass spectrometry (HR-LCMS) analyses were performed on the ethyl acetate extract from both the aboveground and belowground parts. These analyses were conducted at the Sophisticated Analytical Instrument Facility (Saifi et al., 2024), IIT Bombay, Mumbai, India. The HR-LC/MS analysis was carried out using an Agilent 1290 Infinity ultra-high performance liquid chromatography (UHPLC) system coupled with a 1260 Infinity Nano HPLC with Chipcube and 6550 iFunnel Q-TOFs (Agilent Technologies, Santa Clara, CA, United States). The process involved direct infusion mass analysis (MS, MS/MS) with both ESI positive and negative mode ionization. The mass range for the acquisition was set between 50 and 3,200 amu, with a mass precision of less than 1 ppm and a scanning rate of one spectrum per second (Rafiq et al., 2022).

### Bacterial strains and culture

Council of Scientific and Industrial Research, Institute of Microbial Technology (CSIR-IMTech) in Chandigarh, Punjab India furnished the bacterial strains for the study. *Bacillus subtilis, Staphylococcus aureus, Escherchia coli* and *Proteus vulgaris* were used for the current research. The bacterial strain underwent sub-culturing on Mueller Hinton broth media. A 100 μL inoculum of 0.5 Mac-Farland standard was obtained from the freshly prepared bacterial suspension for each bacterial strain and inoculated on molten Mueller Hinton agar in culture tubes.

### Antibacterial activity

The antibacterial activities of different extracts of *B. amplexicaulis* against selected bacterial strains were assessed using the agar well diffusion method given by Perez and Anesini (1994). Ethyl acetate and methanolic extracts were analyzed for their antibacterial activity as they had higher concentrations of the phytochemicals (Phenolic, flavonoid, and tannin) in comparison to the petroleum ether and aqueous extracts. The previously prepared cultured tubes were mixed by gently rubbing them between hands, and the contents were poured into 90 mm sterile Petri plates separately, allowing them to solidify under laminar airflow. Wells were created using a 5 mm standard Cork borer, after which 50 μL of each plant extract, dissolved in DMSO, was added to the respective wells. Kanamycin (50 mg/mL) was used as a positive control, while DMSO (5%) acted as a negative control. The sealed plates were incubated at 37°C ± 1°C for 24 h, and the antibacterial activity was evaluated by measuring the inhibition zones using a standard scale. The results were expressed as mean ± SD from three independent experiments.

The minimum inhibitory concentration (MIC), representing the lowest concentration at which bacterial growth is absent, was determined using the macro dilution method outlined by Jonathan and Fasidi, (2003). Stock solutions were used to prepare the samples through the serial dilution method. Each diluted sample was thoroughly mixed with 20 mL of sterile, molten Muller Hinton agar, and then poured into 90 mm Petri plates. After cooling under laminar air flow, the plates were streaked with 0.5 McFarland standard inoculums (10 μL) of the tested bacterial strains and were incubated at 37°C ± 1°C for 24 h.

### Fungal strains and culture

The Council of Scientific and Industrial Research – Institute of Microbial Technology (CSIR-IMTech) in Chandigarh, Punjab, India, provided the microbial strains for this study. The fungal species included *Pencillium notatum, Aspergillus niger, and Aspergillus flavus*. All of the fungal strains were maintained and cultivated on the Potato Dextrose Agar (PDA) medium.

### Antifungal activity

The antifungal activity of different concentrations of *B. amplexicaulis* rhizome extracts (ethyl acetate and methanol) was evaluated against, *A. niger*, *A. flavus,* and *Penicillium notatum*. Ethyl acetate and methanolic extracts were further analyzed for their antifungal activity as they had higher concentrations of the phytochemicals (Phenolic, flavonoid, and tannin) in comparison to the petroleum ether and aqueous extracts. The poisoned food method was employed, using plant extract concentrations of 200, 400, and 600 μg/mL (Mohana and Raveesha, 2007). The inhibition of mycelial growth was calculated as a percentage using a specific formula. Minimum Inhibitory Concentrations of the plant extracts were determined using a micro-broth dilution procedure, following standard methodologies, to assess antifungal activity against various fungal species. Clotrimazole (5 mg/mL) was taken as a positive control while DMSO (5%) acted as a negative control.

The lowest dosage at which no noticeable growth appears in the wells (showing 80%–100% inhibition) upon visual inspection is known as the minimum inhibitory concentration of an antifungal medication. To ascertain the MIC for each extract, the protocol outlined by Wiegand et al., 2008, was followed. Following this, the plates were incubated at 37°C for 24–48 h. MIC was determined by examining the plates for fungal growth. After 48 h of incubation, MIC values were determined by identifying the lowest extract concentration at which negligible fungal growth was observed. To evaluate the antifungal activity against the aforementioned fungal species, plant extracts were used at doses ranging from 150 to 300 μg/mL.

### *In silico* antimicrobial activity

#### Pharmacokinetic studies

The structure of phyto-compounds was drawn by using ChemDraw 23.0 software and assessed for drug-likeliness properties using SwissADME software http://www.swissadme.ch/ (Daina et al., 2017). Drug likeness was filtered using Lipinski’s rule of five (Lipinski, 2004). Inactive status was assigned to constituents who met less than three requirements.

#### Protein preparation

The target proteins of bacteria and fungi that are essential to their life cycles were chosen for this study. These include (A) Penicillin-binding protein (2C5W) (B) DNA Gyrase (3U2D) (C) Elongation factor (EF-Tu) (1TUI) (D) Sterol 14-alpha demethylase (5TZ1) (E) Fungal 1,3-beta-glucan synthase (8JZN) (F) Lanosterol 14-alpha demethylase (4LXJ). The RCSB PDB library was consulted to obtain their protein structure. The PDB files were then saved (Berman et al., 2000). Subsequently, these proteins were processed for molecular docking using AutoDock version 4.2.1 software. The proteins were refined by removing water molecules, unnecessary chains, and heteroatoms, introducing hydrogen atoms and charges (Kollman charges), and converting them into pdbqt files. The Molecular Operating Environment (MOE) 2019.0102 (Deeth et al., 2005) software was used to identify active docking sites, and grid box dimensions were set appropriately. The protein pdbqt files did not include co-crystallized ligands (Trott and Olson, 2010).

#### Ligand preparation

The bioactive ligand molecules were sourced from PubChem in 3D Standard Data Format (3D SDF) (Wang et al., 2009). To convert these ligands to Protein Data Bank (PDB) format PyMol (Schrodinger, 2015) was used, which was then put into AutoDock Tools for further processing. Rotational bonds were identified and adjusted, Gasteiger charges were applied, and non-polar hydrogen atoms were combined.

#### Virtual screening analysis

Virtual screening of compounds uses computer-based approaches to find novel ligands based on the complementarity and binding of their 3D structure with the target protein’s active site was used to filter 57 chemicals downloaded from the PubChem database using the ADME and Lipinski criteria. This reduced the total number of compounds to 23. After filtering according to their toxicity criteria, virtual screening of 23 compounds was done using the PyRx https://pyrx.sourceforge.io/ (Dallakyan and Olson, 2014) and Auto Dock Vina software https://pubmed.ncbi.nlm.nih.gov/19499576/ (Trott and Olson, 2010). Based on their binding score, the first compounds 23 were selected for the second phase of molecular docking. The top compounds from virtual screening were further docked using the AutoDock tool in the second screening phase. Table 7, shows the set of final 4 compounds selected by highest binding energy and toxicity analysis, and the top one compound (Funtumine) was selected for further evaluation using an MD simulation study at 100 nanoseconds (ns).

#### Molecular docking analysis

Using precise grid box dimensions, AutoDock version 4.2.1 was used to perform a molecular docking analysis of the top compound (Funtumine). Following the Lamarckian Genetic Algorithm (Olğaç et al., 2017) technique, docking was carried out. Discovery Studio Visualizer 2025 was used to compute the binding affinities and analyze the interactions between amino acids and docked poses (Trott and Olson, 2010).

#### Molecular dynamic (MD) simulation studies

Bacterial DNA gyrase (PDB ID: 3U2D) protein MD simulations, in conjunction with ligand (Funtumine) and native ligand, were executed using vs. 2020.1 Desmond simulation engine. The simulations were conducted at a physiological temperature of 37°C, employing a clear model solvent with SPC molecules of water. The SPC water model can capture the essential features of water-protein and water-ligand interactions, such as hydrogen bonding and electrostatic interactions. By solvating the protein-ligand complex with SPC water molecules, we have studied the conformational changes, binding affinities, and dynamic behavior of the complex in a more realistic setting. The OPLS-2005 force field was employed with a periodic boundary solvation box measuring 10 × 10 × 10 Å. A 0.15 M NaCl solution was added to simulate natural biological conditions, and Na^+^ ions were introduced to neutralize the system. OPLS_2005 force field particularly suitable for studying the conformational behavior of proteins and nucleic acids, as well as their interactions with small molecules (Jorgensen and Tirado-Rives, 1988). Initially, the system underwent a stabilization phase of 10 ns in the NVT ensemble, aimed at equilibrating the protein-ligand complexes. Following this, a 12-ns equilibration and minimization were carried out within the NPT ensemble. The NPT ensemble was regulated by the Nose-Hoover chain coupling method, which maintained a temperature with relaxation times set at 1.0 ps, and pressure control was fixed at 1 bar throughout the run. The Martyna-Tuckerman-Klein chain coupling barostat method with a relaxation period of 2 ps was employed for pressure regulation. Long-range electrostatic interactions were computed through the Ewald method with particle mesh, while Coulomb interactions were restricted to a 9 Å cut-off. Bonded forces were calculated for each trajectory using the RESPA integrator with a time step of 2 fs. The production phase of the simulation extended for a total of 100 nanoseconds (ns). The system’s stability during the simulation was evaluated using multiple parameters, including the SASA, Radius of Gyration (Rg), Root Mean Square Fluctuation (RMSF), Root Mean Square Deviation (RMSD), Dynamical Cross-Correlation Maps (DCCM), Principal Component Analysis.

#### Binding free energy analysis

In this study, the binding affinities of ligand-protein complexes were analyzed using the Generalized Born Surface Area (MM/GBSA) technique, integrated with molecular mechanics. This method thoroughly evaluates binding free energies by considering molecular interactions and the impact of solvation. Binding free energy calculations via MM/GBSA were performed with the Python-based script which processed the final 50 frames from the simulation trajectory using a single-step sampling approach to enhance the precision of energy assessments. MM-GBSA computes binding free energy (ΔGbind) through an additive framework, accounting for various energetic factors.

The components contributing to the binding free energy included:

• Electrostatic Interactions: Forces between charged atoms within the system.
• Hydrogen Bonding: Stabilizing effects created when hydrogen atoms form bridges with electronegative atoms.
• Van der Waals Forces: Weak attractive forces occurring between nonpolar regions.
• Covalent Bonding: Strong bonds formed through shared electron pairs between atoms.
• Intra-molecular Interactions: Forces acting within the molecule itself.
• Solvation Energy: The energetic changes due to protein solvation.
• Hydrophobic Effects: Influences arising from the interaction between the ligand and non-polar regions.
• Ligand-specific Energies: Energetic properties intrinsic to the ligand itself $\Delta G_{\text{bind}}=\Delta \text{GMM}+\Delta G_{\text{solv}}-\Delta \text{GSA}$ formula used.

Here’s a breakdown of the terms:

• ΔG_bind_: The overall binding free energy of the ligand-protein complex.
• ΔGMM: The total molecular mechanics energy of the protein-ligand system in isolation, reflecting their inherent interactions without any external influences.
• ΔG_solv_: This represents the difference in solvation energies, determined by subtracting the combined solvation energies of the unbound receptor and ligand from the solvation energy of the ligand-protein complex in its bound state.
• ΔGSA: The change in surface area energies between the ligand and the protein accounts for the surface area exposure changes upon binding.

This comprehensive analysis provides insights into how different molecular components contribute to the binding affinity of the ligand-protein complexes, aiding in understanding their interaction dynamics and stability.

### Statistical analysis

The outcomes for each experiment are displayed as mean ± SD (standard deviation). Parallel studies were conducted to evaluate each of the experiments listed and to compare the results presented in the tables. Various statistical analyses were performed for each test. SPSS 23 (SPSS Inc., Chicago, IL, United States) was utilized for analysis of variance (ANOVA) with Tukey’s test for multiple comparisons in the antibacterial and antifungal assays. Origin Pro 2021 was employed for pairwise comparisons and other correlation plots.

## Results

### Qualitative phytochemical analysis

Using standard techniques, the phytochemical examination of different portions of *B. amplexicaulis* in various solvents verified the presence of phenolics, flavonoids, glycosides, tannins, terpenes, alkaloids, and saponins (Table 1).

**TABLE 1:** Qualitative tests for phytochemicals of *B. amplexicaulis* in different solvents and plant parts (P.E. Petroleum ether, E.A. Ethyl acetate, METH. Methanol, AQ. Aqueous; - absent, + mildly present, ++ present, +++ Strongly present).

Phytochemical	Chemical test	P. E	E. A	METH	AQ	P. E	E. A	METH	AQ
		Aboveground parts				Below ground parts			
Alkaloids	Wagner’s	+	+	+	++	+	+	+	++
Phenolics	Ferric chloride	-	+	+++	++	-	++	+++	+
Flavonoids	Alkaline	-	++	++	+	-	++	+++	+
Tannins	Ferric chloride	-	-	-	-	-	+++	++	-
Terpenes	Salkowski’s	-	-	++	+	-	-	++	+
Quinones	Hydrochloric acid	-	-	++	+	+	+	++	+

### Quantitative analysis of phytochemicals

The total phenolic content (TPC) and total flavonoid content (TFC) exhibited significant variation based on the plant part and the solvent utilized. Generally, ethyl acetate extracts showed higher TPC (149.138 ± 1.188 mg GAE/g) and TFC (170.364 ± 4.653 mg rutin/g) compared to other solvents employed. The order of increase in TPC and TFC was observed as follows: petroleum ether > aqueous > methanol > ethyl acetate extracts. Additionally, TPC and TFC levels differed among plant parts, with the highest TPC found in the below-ground parts (rhizomes) compared to the above-ground parts (stems, leaves, and inflorescence). Ethyl acetate extracts of belowground parts contained a higher content of tannins (98.23 ± 0.88 mg GAE/g), whereas aboveground parts had lower amounts (78.815 ± 1.019 mg GAE/g). Tannin content increased as follows: P.E > Ethyl acetate > Methanol > Aqueous extracts in both below and aboveground parts (Table 2).

**TABLE 2:** Quantification of Flavonoids (mg Rutin/g), Phenolics (mg GAE /g), Tannins (mg/ GAE g) in different solvents and plant parts BG-Belowground parts, AG- Aboveground parts.

Phytochemicals	Petroleum ether (P.E)	Ethyl acetate (E.A)	Methanol	Aqueous
Flavonoids (AG)	18.311 ± 0.618^d^	170.364 ± 4.653^a^	64.454 ± 1.442^a^	20.165 ± 0.898^c^
Flavonoids (BG)	34.997 ± 0.714^a^	97.482 ± 1.490^e^	57.038 ± 0.412^ab^	40.421 ± 2.812^a^
Phenolics (AG)	15.267 ± 0.396^b^	109.195 ± 0.605^c^	68.997 ± 1.684^b^	22.858 ± 0.695^e^
Phenolics (BG)	28.799 ± 1.613^a^	149.138 ± 1.188^a^	86.554 ± 0.343^a^	48.073 ± 0.824^a^
Tannins (AG)	13.92 ± 0.56^d^	78.815 ± 1.019^c^	60.892 ± 1.1575^b^	18.6623 ± 0.486^d^
Tannins (BG)	25.52 ± 0.52^a^	98.234 ± 0.88^a^	72.877 ± 0.3923^a^	31.906 ± 1.579^a^

Means labelled with the small letters designate that they vary significantly from each other (Tukey test: P ≤ 0.05) *Mean ± Standard Error.

### Phytochemical composition

**FIGURE 3:**
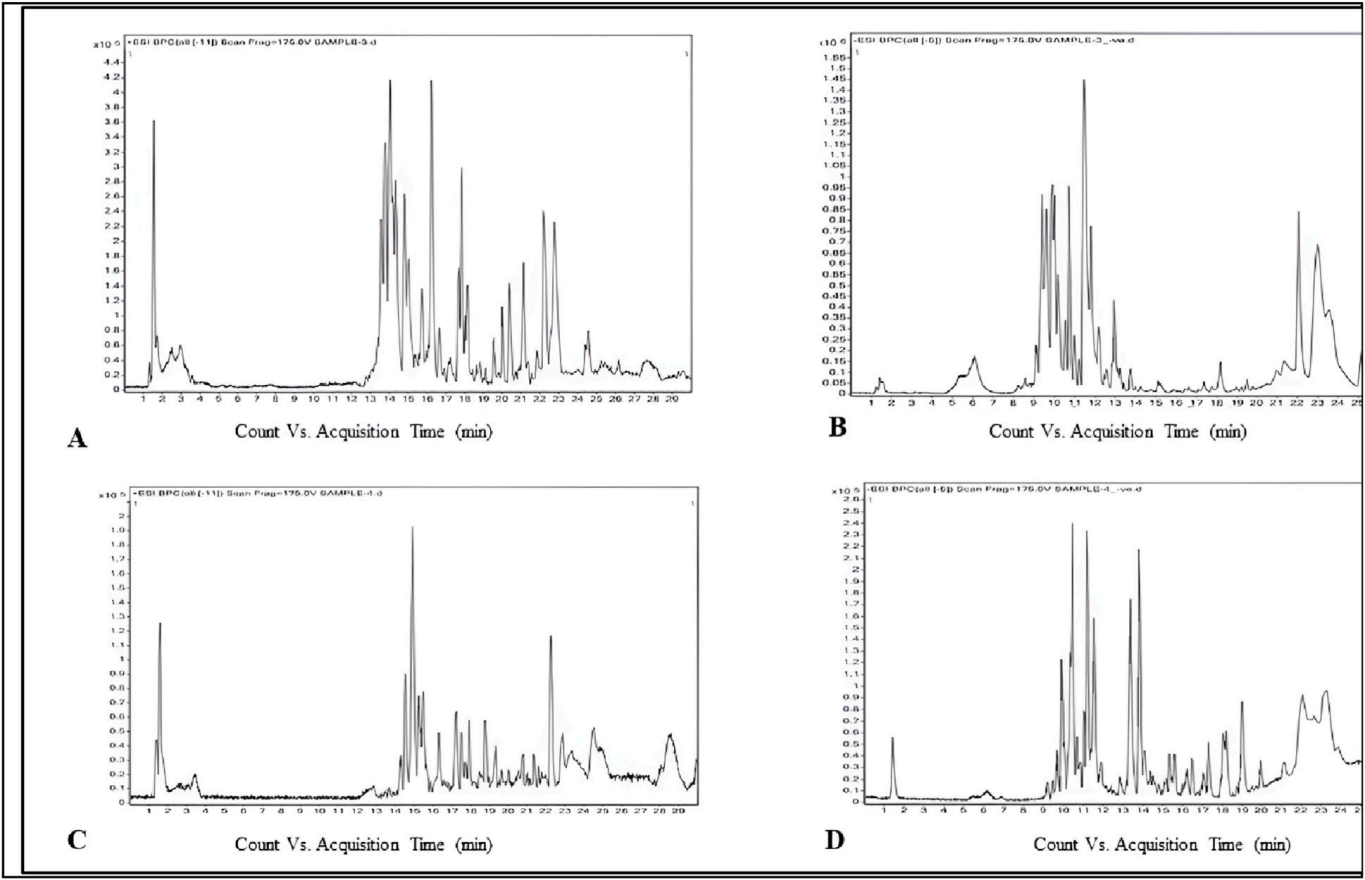
Phytochemicals identified in *B. amplexicaulis* ethyl acetate extract of below ground parts (rhizome) (**(A)** ESI positive mode, **(B)**- ESI negative mode) and above ground parts (leaf, stem, and flower) (**(C)**- ESI positive mode, **(D)**- ESI negative mode) using the HR-LCMS technique.

The phytochemicals in the belowground and aboveground extracts of *B. amplexicaulis* were analyzed and identified using the HR-LCMS approach. The major phenolic and flavonoid compounds identified were Catechin, 3-Hydroxycoumarin, Quercetin, and Paradol, as shown in Table 3. The ethyl acetate extracts from both belowground and aboveground parts displayed 15 and 20 significant peaks in ESI^+ve^ and ESI^−ve^ modes, as illustrated in Figure 3. Previous research by various scholars (Li et al., 2018; Nguyen and Bhattacharya, 2022; Veiko et al., 2023) has already highlighted the antimicrobial properties of Catechin, Quercetin, and other phenolic compounds. Consequently, these compounds may collaborate with other phytochemicals to enhance antimicrobial effects.

**TABLE 3:** (A) Phytochemicals identified in *B. amplexicaulis* ethyl acetate extract of below ground parts (Rhizome) using HR-LC/MS. (B) Phytochemicals identified in *B. amplexicaulis* ethyl acetate extract of Above ground parts (Leaf, stem, and flower) using HR-LC/MS.

S. no	Retn. Time	m/z	Adduct	Compound name	Compound formula	Category/Class	Exact Mass
(A)							
1	1.617	160.096	(M + H)^+^	Medicanine	C H_13_ N O_3_	Alpha amino acids	159.089
2	13.702	579.154	(M + H)^+^	Procyanidin B7	C_30_ H_26_ O_12_	Flavan-3-ols	578.137
3	14.17	355.099	(M + Na)^+^	Leonuriside A	C_14_ H_20_ O_9_	Phenolic glycosides	332.11
4	15.226	303.047	(M + H)^+^	Quercetin	C_15_ H_10_ O_7_	Flavonoids	302.04
5	16.505	515.15	(M + Na)^+^	Globularin	C_24_H_28_O_11_	Flavonoid glycoside	492.161
6	15.81	449.325	(M + H)^+^	Geranyl arabinopyranosylglucoside	C_21_H_36_ O_10_	Terpene glycoside	448.227
7	16.431	344.146	(M + H)^+^	N-trans-Feruloyl-4-Omethyldopamine	C_19_ H_21_N O_5_	Cinnamamides	343.139
8	16.466	314.136	(M + Na)^+^	(S)-Edulinine	C_16_ H_21_ N O_4_	Hydroquinolones	291.147
9	16.505	515.15	(M + H)^+^	2'',6''-Di-O-acetylononin	C_26_ H_26_ O_11_	Isoflavonoid	514.143
10	17.539	373.162	(M + Na)^+^	Arctiopicrin	C_19_ H_26_ O_6_	Sesquiterpene	350.172
11	17.934	557.217	(M + Na)^+^	Sarothralin	C_31_ H_34_ O_8_	Benzophenones	534.228
12	20.786	391.249	(M + Na)^+^	Cortol	C_21_ H_36_ O_5_	Monoterpenoids	368.253
13	24.457	391.249	(M + H)^+^	Icaceine	C_22_ H_33_ N O_4_	Alkaloids	375.241
14	24.896	461.285	(M + H)^+^	Lucidenic acid N	C_27_ H_40_ O_6_	Tetracyclic triterpenoid	460.278
15	25.213	411.17	(M + H)^+^	Bruceine D	C_20_ H_26_ O_9_	Triterpene lactones	410.162
16	9.254	331.046	(M−H)^-^	Patuletin	C_16_ H_12_ O_8_	Flavonol	332.053
17	9.731	423.049	(M + HCOO)^-^	Chlorflavonin	C_18_ H_15_ C_l_ O_7_	Flavone	378.051
18	9.854	191.036	(M−H)^-^	Isoscopoletin	C_10_ H_8_ O_4_	Hydroxycoumarin	192.043
19	11.005	367.066	(M + HCOO)^-^	Gallocatechin-4beta-ol	C_15_ H_14_ O_8_	Flavanoids	322.068
20	11.212	483.138	(M + CH3COO)^-^	Adifoline	C_22_ H_2_0 N_2_ O_7_	Alkaloids	424.124
21	11.942	493.063	(M + HCOO)^-^	Glucobrassicin	C_16_ H_2_0 N_2_ O_9_ S_2_	Isothiocyanate	448.067
22	21.281	315.165	(M−H)^-^	Capillartemisin A	C_19_ H_24_ O_4_	Hydroxycinnamic acid	316.172

### Antibacterial activity

**FIGURE 4:**
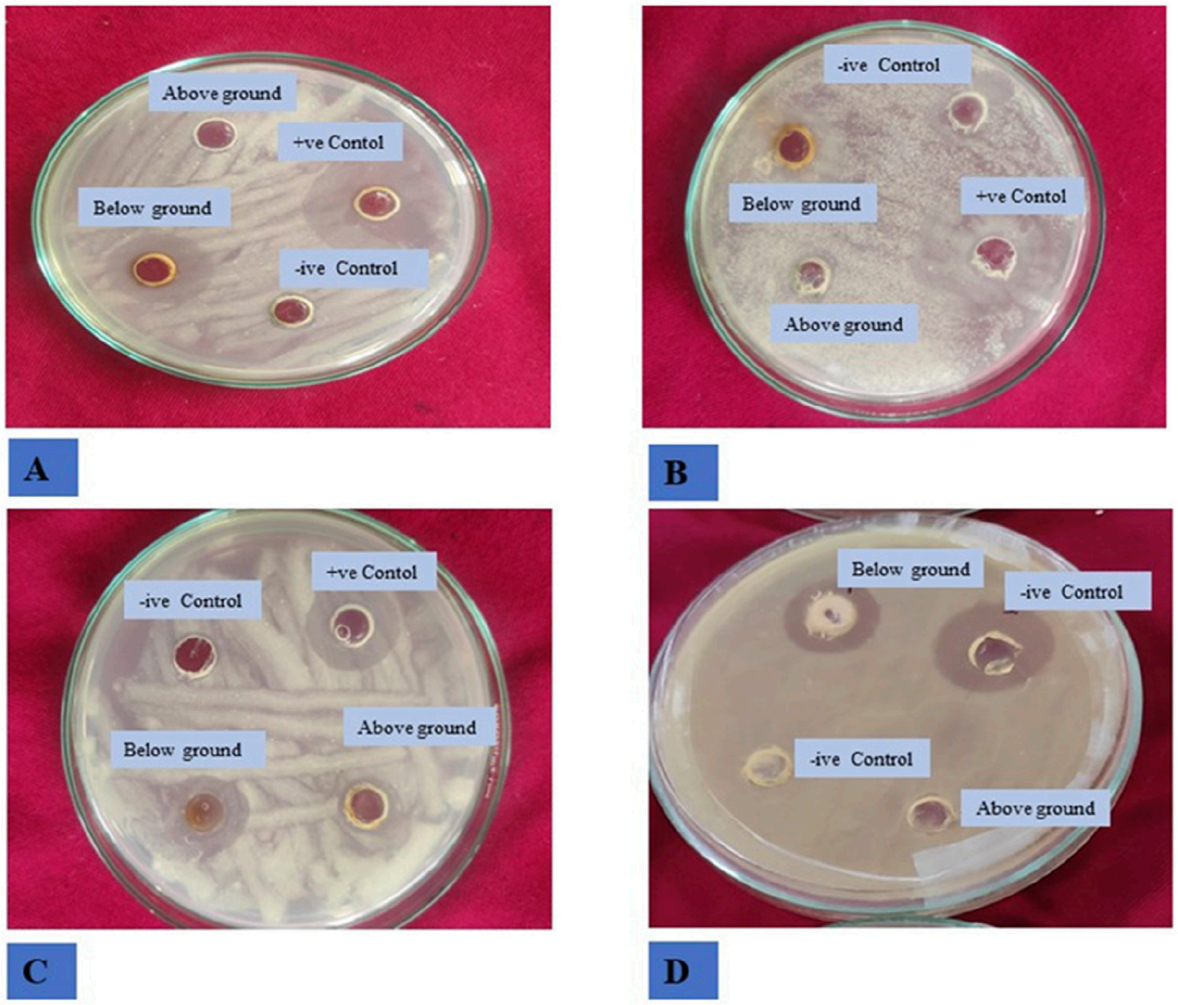
Antibacterial activity of *B. amplexicaulis* against different bacterial strains **(A)** *Bacillus subtilis* **(B)** *Staphylococcus aureus* **(C)** *Proteus vulgaris* **(D)** *Escherichia coli*.

**FIGURE 5:**
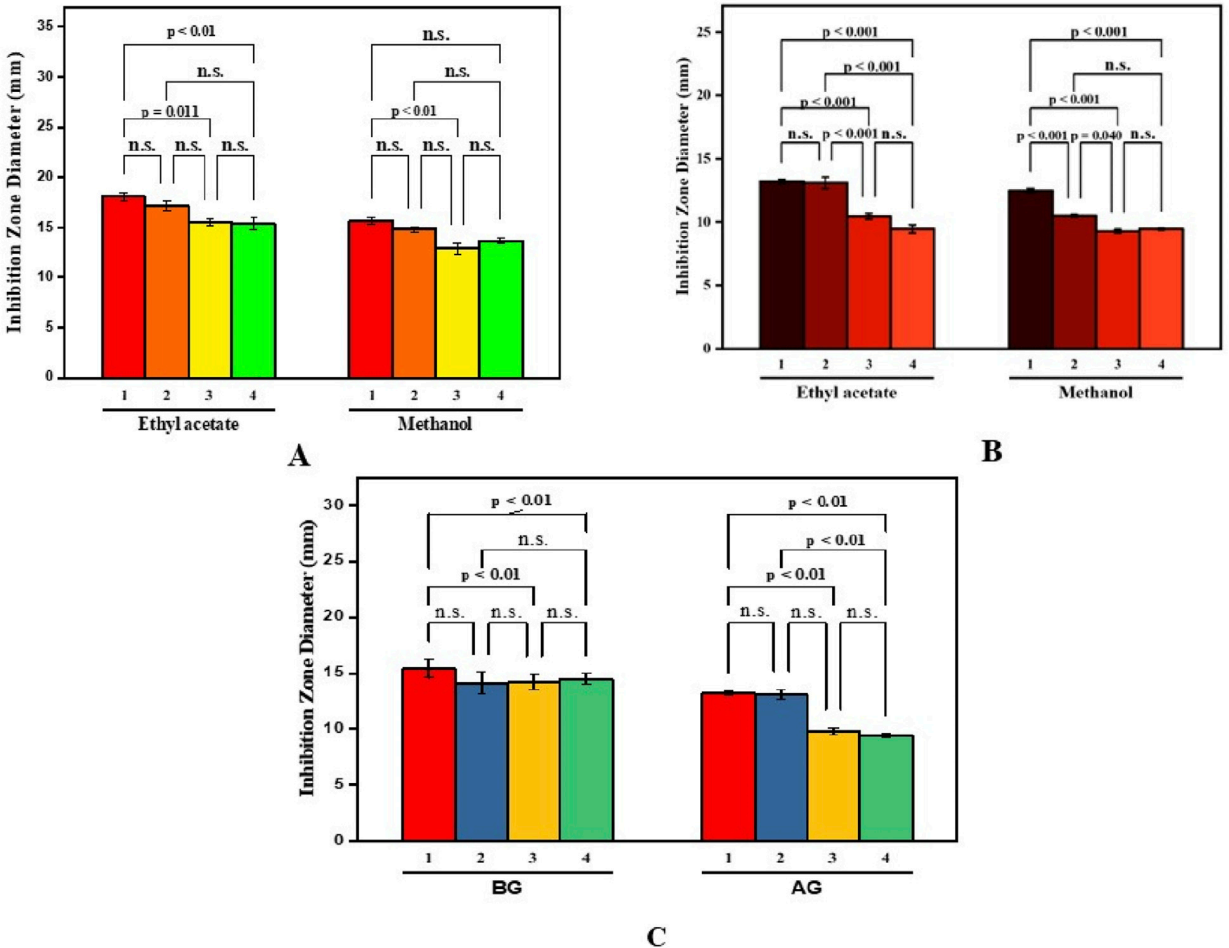
Paired comparison plot showing partwise variation in the antibacterial activity of different solvent extracts of *B. amplexicaulis* (**(A)**-aboveground **(B)**- belowground parts **(C)**- both aboveground and belowground; (1-*Bacillus subtilis* 2-*Staphylococcus aureus* 3-*Escherchia coli* 4-*Proteus vulgaris*). The significance level in antifungal potential between different bacterial strains is denoted as stars (***p ≤ 0.001, **p ≤ 0.01, *p ≤ 0.05), ns, not significant).

The study investigated the antibacterial effects of crude extracts from *B. amplexicaulis*, including ethyl acetate and methanol extracts., against two Gram-positive bacterial strains (*B. subtilis and S. aureus*) and two Gram-negative bacterial strains (*E. coli and P. vulgaris*). Various concentrations of the extracts ranging from 400 μg/mL to 1,000 μg/mL were tested against these bacterial strains. Kanamycin antibiotic served as the positive control, and 10% aqueous DMSO (dimethyl sulfoxide) was used as the negative control. The inhibitory effects of the extracts were observed to be concentration and solvent-polarity-dependent. The antimicrobial activity was quantified using the Inhibition Zone Diameter (IZD) measured in millimeters. Both ethyl acetate extract and methanolic extract from *B. amplexicaulis* demonstrated inhibitory effects against all four bacterial strains (*Escherichia coli, P. vulgaris, S. aureus, B. subtilis*) as shown in (Table 4; Figure 4). Ethyl acetate extracts (belowground parts) displayed the broadest Inhibition Zone Diameter (IZD) at 18.07 ± 0.38 mm against *B. subtilis* at a concentration of 1,000 μg/mL, and the lowest IZD at 5.50 ± 0.39 mm against *P. vulgaris* at 400 μg/mL (aboveground parts). Interestingly, methanolic extracts (belowground parts) showed the highest IZD at 15.69 ± 0.32 mm against *B. subtilis* at 1,000 μg/mL concentration, and the lowest IZD at 5.30 ± 0.15 mm against *P. vulgaris* at 400 μg/mL (aboveground parts). Both ethyl acetate and methanolic extracts from the belowground parts demonstrated active antibacterial properties against various tested bacterial strains, including *S. aureus, E. coli, and P. vulgaris*, with IZD values of 17.13 ± 0.48 mm, 15.55 ± 0.34 mm, and 13.42 ± 0.16 mm, respectively, at a concentration of 1,000 μg/mL. Overall, ethyl acetate extracts exhibited superior inhibition potential compared to methanolic extracts in both belowground and aboveground parts. The results also indicate that the belowground parts of the plant exhibit higher inhibition potential compared to the aboveground parts (Figures 5A–C). The Minimum Inhibitory Concentration (MIC) values for ethyl acetate extracts of belowground parts ranged from 100 μg/mL to 300 μg/mL, whereas the MIC values for methanolic extracts ranged from 200 μg/mL to >400 μg/mL (Table 4).

**TABLE 3:** **(B)** Phytochemicals identified in *B. amplexicaulis* ethyl acetate extract of Above ground parts (Leaf, stem, and flower) using HR-LC/MS.

S. no	Retn. Time	m/z	Adduct	Compound Name	Compound formula	Category/Class	Exact Mass
(B)							
1	15.254	290.08	(M + H)^+^	Catechin	C_15_ H_14_ O_6_	Polyphenols	291.085
2	15.287	432.102	(M + H)^+^	Emodin 8-glucoside	C_21_ H_20_ O_10_	Hydroxyanthraquinones	433.11
3	1.656	159.088	(M + H)^+^	Medicanine	C_7_ H_13_ N O_3_	Alpha amino acids	160.096
4	14.529	332.11	(M + Na)^+^	Leonuriside A	C_14_ H_20_ O_9_	Phenolic glycosides	355.099
5	14.576	112.054	(M + H)^+^	1,4-Cyclohexanedione	C_6_ H_8_ O_2_	Tetrahydroquinone	113.058
6	14.59-14.885	162.031	(M + H)^+^	3-Hydroxycoumarin	C_9_ H_6_ O_3_	Phenolic	163.038
7	14.654	268.095	(M + Na)^+^	Disperse Blue 1	C_14_ H_12_ N_4_ O_2_	Aminoanthraquinone	291.084
8	14.849	317.267	M + Na)^+^	Funtumine	C_21_ H_35_ N O	Alkaloid	340.257
9	14.888	448.097	(M + H)^+^	6-C-Galactosylluteolin	C_21_ H_20_ O_11_	Flavone C-glycoside	449.104
11	15.031-15.527	614.183	(M + Na)^+^	Catechin 5,4'-di-O-beta-D-glucopyranoside	C_27_ H_34_ O_16_	Flavonoid	637.172
12	15.075	556.175	(M + Na)^+^	7-Dehydrologanin tetraacetate	C_25_ H_32_ O_14_	Terpene glycoside	579.165
13	15.281	448.097	(M + H)^+^	6-C-Galactosylluteolin	C_2_1 H_20_ O_11_	Flavone C-glycoside	449.104
14	15.337	432.102	(M + H)^+^	Apigenin 7-glucoside	C_21_ H_20_ O_10_	Flavone	433.11
15	15.367	440.13	(M + Na)^+^	Ginkgolide C	C_20_ H_24_ O_11_	Diterpenoids	463.119
16	15.576	284.126	(M + Na)^+^	2-Phenylethyl beta-D- glucopyranoside	C_14_ H_20_ O_6_	Glycoside	307.115
17	15.684	302.041	(M + H^)+^	Quercetin	C_15_ H_10_ O_7_	Flavonoids	303.05
18	15.875	650.18	(M + H)^+^	Hesperetin 5,7-O- diglucuronide	C_30_ H_34_ O_16_	Flavonoids	651.188
19	16.054	364.191	(M + H)^+^	Blumealactone A	C_20_ H_28_ O_6_	Terpene lactone	365.194
20	16.361	132.094	(M + H)^+^	Tetralin	C_10_ H_12_	Phenylpropanoic acids	133.101
21	16.673	313.13	(M + H)^+^	N-Feruloyltyramine	C_18_ H_19_ N _O4_	Alkaloid	314.137
22	17.208	350.205	(M + H)^+^	4,4-Difluoropregn-5-ene-3,20- dione	C_21_ H_28_ F_2_ O_2_	Steroid	351.212
23	17.503	692.36	(M + Na)^+^	Bismahanine	C_46_ H_48_ N_2_ O_4_	Carbazole alkaloid	715.351
26	18.179	310.214	(M + Na)^+^	Sterebin A	C_18_ H_30_ O_4_	Bisnorditerpenoids	333.203
27	18.782	276.172	(M + H)^+^	Onchidal	C_17_ H_24_ O_3_	Sesquiterpenoid	277.179
28	18.784-19.476	310.213	(M + Na)^+^	Sterebin A	C_18_ H_30_ O_4_	Sesquiterpenoids	333.202
29	19.152	363.312	(M + H)^+^	Terminaline	C_23_ H_4_1 N O_2_	Steroidal alkaloid	364.319
31	19.705	334.213	(M + Na)^+^	12-oxo-LTB4	C_20_ H_30_ O_4_	Benzylisoquinoline	357.202
32	20.487	280.073	(M + Na)^+^	Methoxybrassenin A	C_1_3 H_16_ N_2_ O S_2_	Alkaloid	303.062
33	20.78	316.203	(M + Na)^+^	Furanojaponin	C_2_0 H_28_ O_3_	Sesquiterpenoids	339.192
34	20.954	292.204	(M + Na)^+^	[7]-Paradol	C_18_ H_28_ O_3_	Phenols	315.194
35	21.186	476.279	(M + Na)^+^	Lucidenic acid H	C_27_ H_40_ O_7_	Triterpenoid	499.268

### Antifungal activity

**FIGURE 6:**
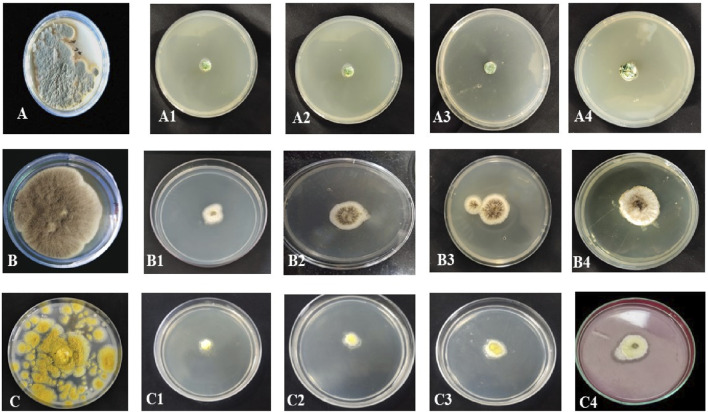
Antifungal activity of *B. amplexicaulis* against different fungal species **(A)** Negative control Penicillium notatum **(B)** Negative control *Aspergillus niger* **(C)** Negative control *Aspergillus flavus*, (A1,A2) Inhibition by ethyl acetate (600 μg/mL) and methanol (600 μg/mL) extracts of belowground parts against *P. notatum*, (A3,A4) Inhibition by ethyl acetate (600 μg/mL) and methanol (600 μg/mL) extracts of aboveground parts against *P. notatum*, (B1,B2) Inhibition by ethyl acetate (600 μg/mL) and methanol (600 μg/mL) extracts of belowground parts against *A. niger* (B3,B4) Inhibition by ethyl acetate (600 μg/mL) and methanol (600 μg/mL) extracts of aboveground parts against *A. niger*, (C1,C2) Inhibition by ethyl acetate (600 μg/mL) and methanol (600 μg/mL) extracts of belowground parts against *A. flavus* (C3,C4) Inhibition by ethyl acetate (600 μg/mL) and methanol (600 μg/mL) extracts of aboveground parts against *A. flavus.*

**FIGURE 7:**
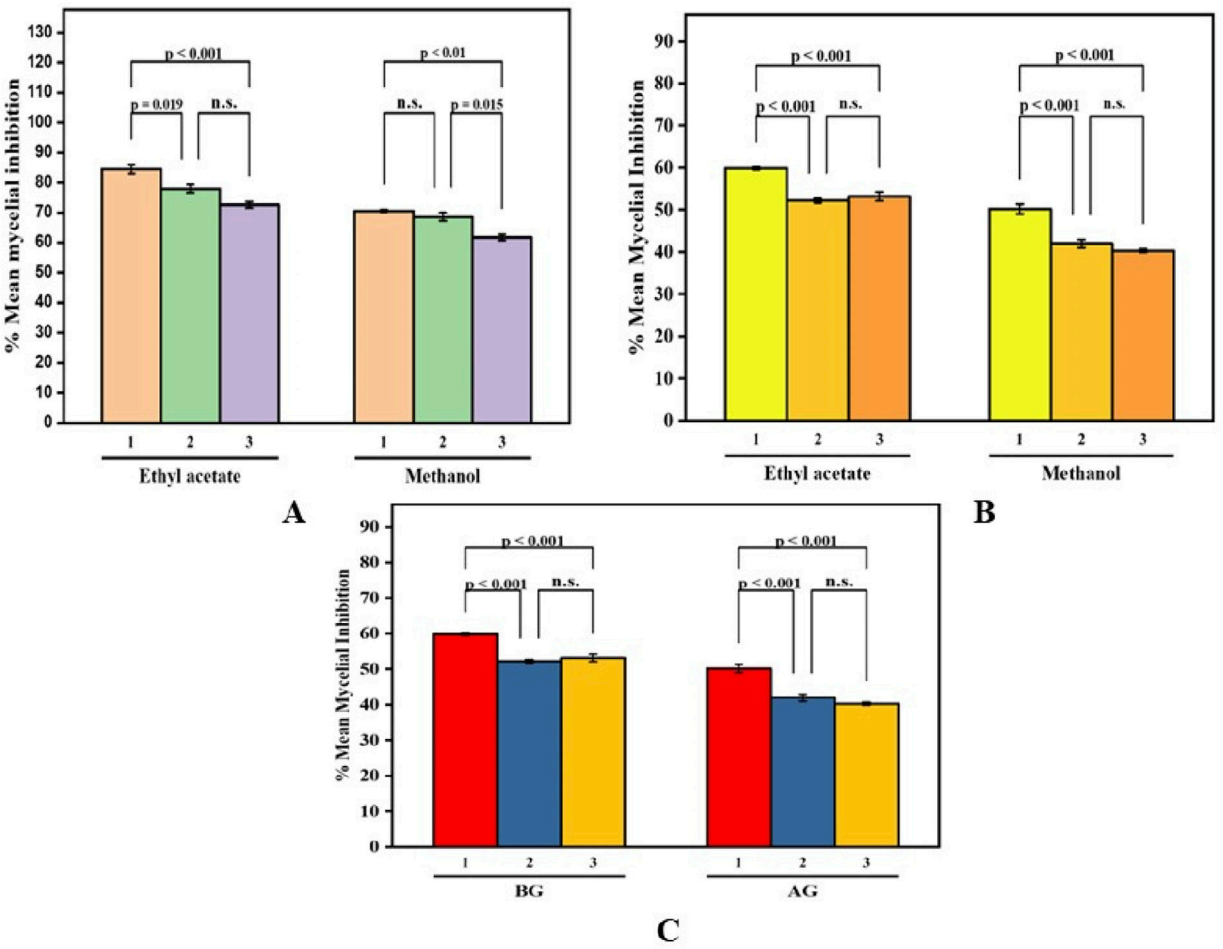
Paired Comparison plot showing part wise variation in the antifungal activity of different extracts of *B. amplexicaulis* (**(A)**-Aboveground **(B)**- Belowground parts **(C)**- Both aboveground and belowground; 1- *Penicillium notatum* 2*- Aspergillus niger* 3- *Aspergillus flavus*) The significance level in antifungal potential between different fungal strains is denoted as stars (***p ≤ 0.001, **p ≤ 0.01, *p ≤ 0.05), ns, not significant).

The inhibitory effects of different doses of *B. amplexicaulis* extracts against *A. flavus*, *A. niger*, and *Penicillium notatum* were evaluated using the poisoned food technique, with the results detailed in Table 5. In the poisoned food technique, the test fungi formed colonies with a significantly smaller average diameter compared to those in the control plates, highlighting the antifungal potential of the extracts (Table 5; Figure 6). The degree of inhibition was directly proportional to the extract concentration. The fungi exhibited varying levels of sensitivity to the extracts; higher extract concentrations led to greater inhibition of fungal mycelial growth. The findings reveal that the ethyl acetate extracts from the belowground parts exhibited the maximum percentage inhibition against *P. notatum*, while the lowest was observed against *A. niger*. Ethyl acetate extracts of belowground parts at a concentration of 600 μg/mL showed the highest inhibition of mycelial growth against *P. notatum*, resulting in an 84.56% ± 2.56% reduction in mycelial growth compared to the control. Conversely, the lowest inhibition of mycelial growth was observed with 200 μg/mL methanol extracts of aboveground parts against *A. flavus*, resulting in an inhibition of 26.18% ± 2.58% in mycelial growth compared to the control. Belowground plant parts have greater inhibitory potential when compared with the aboveground parts. The ethyl acetate and methanolic extracts of belowground parts also exhibited effective antifungal potential against other tested fungal strains, such as *A. niger*, *A. flavus.* The corresponding % mycelial inhibition values were 72.61% ± 1.93% and 77.95% ± 2.41% respectively. The minimum inhibitory concentration for ethyl acetate extracts varied from 190 to 320 μg/mL, whereas the MIC for methanolic extracts ranged between 260 and 350 μg/mL (Table 5). In summary, ethyl acetate extracts demonstrated a stronger inhibition potential than methanolic extracts in both belowground and aboveground plant parts. The findings also reveal that belowground parts have a higher inhibition potential than aboveground parts (Figures 7A–C).

**TABLE 4:** Antibacterial activity (Zone of inhibition (mm)) of below ground and above ground extracts of *B. amplexicaulis* in different solvents.

	Ethyl acetate (belowground)			MIC (µg/mL)	Ethyl acetate (aboveground)			MIC (µg/mL)
Strain	400 (µg/mL)	700 (µg/mL)	1,000 (µg/mL)		400 (µg/mL)	700 (µg/mL)	1,000 (µg/mL)	
*Bacillus subtilis*	12.77 ± 0.32^a^	14.14 ± 0.33^ab^	18.07 ± 0.38^a^	100	9.46 ± 0.45^a^	10.01 ± 0.86^a^	13.21 ± 0.29^a^	300.00
*Staphylococcus aureus*	10.19 ± 0.16^bc^	13.88 ± 0.55^ab^	17.13 ± 0.48^ab^	100	7.36 ± 0.07^bc^	10.44 ± 0.29^a^	13.09 ± 0.79^a^	300.00
*Escherichia coli*	10.93 ± 0.35^b^	14.29 ± 1.00^a^	15.55 ± 0.34^bc^	200	7.00 ± 1.14^c^	8.47 ± 0.27^b^	10.44 ± 0.38^b^	400.00
*Proteus vulgaris*	8.96 ± 0.45^c^	10.29 ± 0.16^c^	13.42 ± 0.16^d^	300	5.50 ± 0.39^d^	7.99 ± 0.49^bc^	9.33 ± 0.21^b^	>400

	Methanol (belowground)			MIC (µg/mL)	Methanol (aboveground)			MIC (µg/mL)
Strain	400 (µg/mL)	700 (µg/mL)	1,000 (µg/mL)		400 (µg/mL)	700 (µg/mL)	1,000 (µg/mL)	
*Bacillus subtilis*	10.07 ± 0.15^a^	13.1 ± 0.44^a^	15.69 ± 0.32^a^	200	8.37 ± 0.03^a^	9.75 ± 0.22^a^	12.48 ± 0.16^a^	300
*Staphylococcus aureus*	9.48 ± 0.20^a^	12.79 ± 0.07^a^	14.77 ± 0.28^ab^	200	6.66 ± 0.23^b^	8.88 ± 0.19^ab^	10.47 ± 0.07^b^	>400
*Escherichia coli*	8.21 ± 0.063^bc^	9.80 ± 0.25^bc^	12.85 ± 0.51^c^	300	5.33 ± 0.16^c^	7.26 ± 0.14^c^	9.26 ± 0.14^c^	>400
*Proteus vulgaris*	6.53 ± 0.09^d^	8.47 ± 0.09^c^	10.55 ± 0.38^d^	400	5.30 ± 0.15^c^	6.25 ± 0.09^d^	7.15 ± 0.10^d^	>400

**TABLE 5:** Antifungal activity (percentage mycelial inhibition) of below ground and above ground extracts of *B. amplexicaulis* in different solvents.

	Ethyl acetate (belowground)			MIC (µg/mL)	Ethyl acetate (aboveground)			MIC (µg/mL)
Strain	200 µg/mL	400 µg/mL	600 µg/mL		200 µg/mL	400 µg/mL	600 µg/mL	
*Pencillium notatum*	53.33 ± 2.04^a^	74.39 ± 2.82^a^	84.56 ± 2.56^a^	190	41.55 ± 0.58^a^	52.25 ± 1.06^a^	59.85 ± 0.54^a^	300
*Aspergillus niger*	40.14 ± 0.56^b^	64.17 ± 3.01^b^	77.95 ± 2.41^ab^	220	38.55 ± 0.78^b^	45.77 ± 2.55^b^	53.15 ± 1.77^b^	310
*Aspergillus flavus*	44.27 ± 3.09^b^	60.07 ± 2.66^b^	72.61 ± 1.93^b^	250	35.19 ± 0.89^c^	44.36 ± 2.81^b^	52.19 ± 0.90^b^	320

	Methanol (belowground)			MIC (µg/mL)	Methanol (aboveground)			MIC (µg/mL)
Strain	200 µg/mL	400 µg/mL	600 µg/mL		200 µg/mL	400 µg/mL	600 µg/mL	
*Pencillium notatum*	44.26 ± 3.10^a^	55.49 ± 1.04^a^	70.6 ± 0.58^a^	260	32.29 ± 1.17^a^	41.12 ± 1.09^a^	50.23 ± 2.00^a^	330
*Aspergillus niger*	41.32 ± 0.85^a^	49.29 ± 1.90^b^	61.74 ± 1.89^c^	300	27.81 ± 2.07^ab^	33.06 ± 1.29^b^	40.33 ± 0.79^b^	350
*Aspergillus flavus*	41.85 ± 1.99^a^	51.85 ± 1.52^ab^	68.57 ± 2.31^ab^	270	26.18 ± 2.58^b^	32.63 ± 2.73^b^	39.50 ± 1.62^b^	350

### Relationship between phytochemicals, antibacterial and antifungal activity

The current study revealed antibacterial (IZD) and antifungal (% MI) activity showed a positive correlation with the concentration of phenolics and tannins however with flavonoids a negative correlation is found. This implies that the higher the concentration of the phenolics and tannins better the antibacterial and antifungal activity.

### Pharmacokinetic studies of the identified compounds

To ensure a thorough evaluation of the pharmacological potential of the identified compounds, an extensive assessment of their pharmacological properties was performed using the SwissADME online software (Daina et al., 2017). Table 6 contain the detailed results of these evaluations. The identified compounds were found to be exceptionally adhered well to Lipinski’s rule of five.

**TABLE 6:** ADME/T properties Phytochemicals identified in *B. amplexicaulis* ethyl acetate extract of Above and below ground parts using SwissADME software.

Molecule ID	MW (g/mol)	wLOGP	nHBA	nHBD	Lipinski violations	Bioavailability score	Synthetic accessibility
Medicanine	159.18	−0.85	4	2	0	0.55	1.99
Procyanidin B7	578.52	2.35	12	10	3	0.17	5.29
Leonuriside A	332.30	−1.41	9	5	0	0.55	4.53
N-Feruloyltyramine	313.35	2.37	4	3	0	0.55	2.55
Globularin	492.47	−1.43	11	5	1	0.55	6.12
(S)-Edulinine	291.34	1.22	4	2	0	0.55	3.19
2,6-Di-Oacetylononin	514.48	1.79	11	2	2	0.17	5.51
Capillartemisin A	316.39	3.37	4	3	0	0.56	3.04
Methoxybrassenin A	280.41	3.13	2	0	0	0.55	2.93
Emodin 8-glucoside	432.38	−0.64	10	6	1	0.55	5.06
Medicanine	159.18	−0.85	4	2	0	0.55	1.99
Leonuriside A	332.30	−1.41	9	5	0	0.55	4.53
1,4-Cyclohexanedione	112.13	0.70	2	0	0	0.55	1.29
3-Hydroxycoumarin	162.14	1.50	3	1	0	0.55	2.66
Disperse Blue 1	268.27	0.82	2	4	0	0.55	2.37
Funtumine	317.51	4.56	2	1	1	0.55	4.07
6-C-Galactosylluteolin	448.38	−0.53	11	8	2	0.17	5.04
Catechin5,4'-di-O-beta-D-glucopyranoside	614.55	−3.83	16	11	3	0.17	6.48
7-Dehydrologanin tetraacetate	556.51	0.34	14	0	2	0.11	6.39
Sterebin A	310.43	2.07	4	3	0	0.55	4.98
6-C-Galactosylluteolin	432.38	0.05	10	6	1	0.55	5.12
Apigenin 7-glucoside	440.40	−2.40	11	4	1	0.55	6.48
Ginkgolide C	416.42	−2.49	10	6	1	0.55	5.35

MW: molecular weight; nHBA: Number of hydrogen bond acceptors; nHBD: Number of hydrogen bond donors

### Molecular docking analysis

**FIGURE 8:**
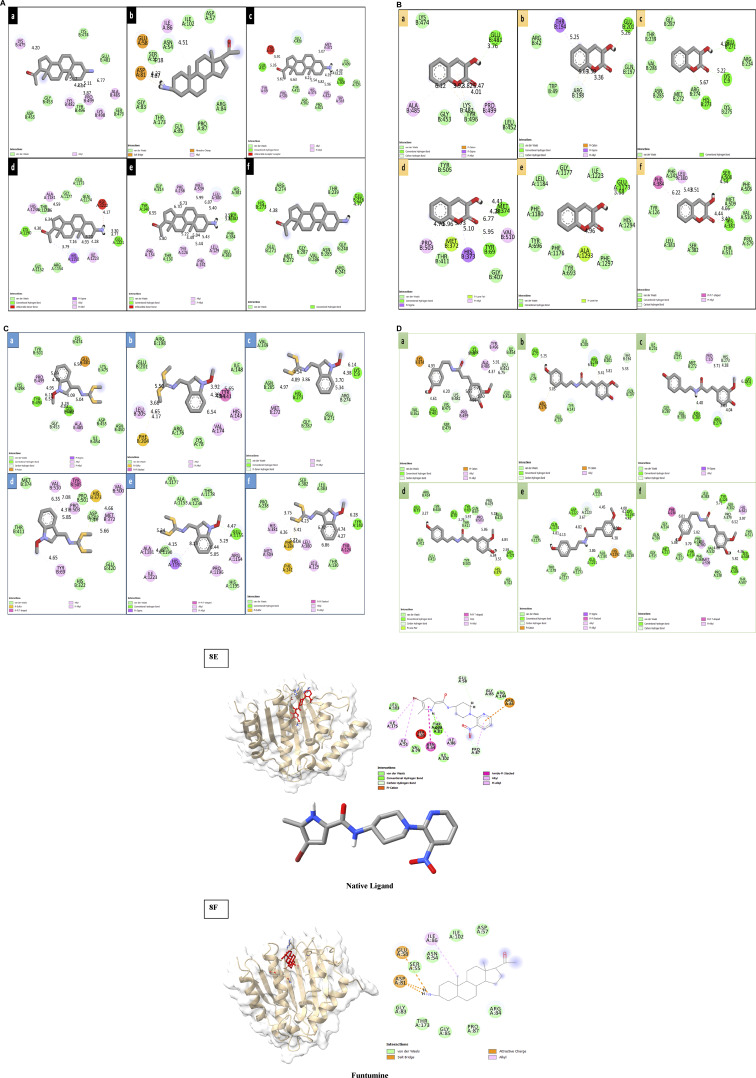
**(A)** 2D diagrams of the compound (Funtumine) against bacterial targets (a–c) and fungal targets (d–f): (a) Penicillin-binding protein (2C5W) (b) DNA Gyrase (3U2D) (c) Elongation factor (EF-Tu) (1TUI) (d) Sterol 14-alpha demethylase (5TZ1) (e) Fungal 1,3-beta-glucan synthase (8JZN) (f) Lanosterol 14-alpha demethylase (4LXJ). **(B)** 2D diagrams of the compound (3-hydroxycoumarin) against bacterial targets (a–c) and fungal targets (d–f): (a) Penicillin-binding protein (2C5W) (b) DNA Gyrase (3U2D) (c) Elongation factor (EF-Tu) (1TUI) (d) Sterol 14-alpha demethylase (5TZ1) (e) Fungal 1,3-beta-glucan synthase (8JZN) (f) Lanosterol 14-alpha demethylase (4LXJ). **(C)** 2D diagrams of the compound (Methoxybrassenin A) against bacterial targets (a–c) and fungal targets (d–f): (a) Penicillin-binding protein (2C5W) (b) DNA Gyrase (3U2D) (c) Elongation factor (EF-Tu) (1TUI) (d) Sterol 14-alpha demethylase (5TZ1) (e) Fungal 1,3-beta-glucan synthase (8JZN) (f) Lanosterol 14-alpha demethylase (4LXJ). **(D)** 2D diagrams of the compound (N-Feruloyl tyramine) against bacterial targets (a–c) and fungal targets (d–f): (a) Penicillin-binding protein (2C5W) (b) DNA Gyrase (3U2D) (c) Elongation factor (EF-Tu) (1TUI) (d) Sterol 14-alpha demethylase (5TZ1) (e) Fungal 1,3-beta-glucan synthase (8JZN) (f) Lanosterol 14-alpha demethylase (4LXJ). **(E,F)**: 3D and 2D diagrams of the Native ligand and Funtumine against bacterial DNA Gyrase (3U2D) protein target.

Following ADME analysis, the identified compounds were selected for virtual screening against the chosen bacterial and fungal protein drug targets. Among these, Funtumine demonstrated the most favorable binding characteristics, including the lowest binding energy, and significant hydrophobic interactions when compared to the top four compounds screened against the microbial targets. These results, illustrated in Figures 8A–F, indicate that Funtumine exhibited the most promising binding score, with strong interactions involving key binding residues, good compatibility with the active site, and optimal bond distances. Specifically, the docking affinity of Funtumine with DNA gyrase was calculated at –8.23 kcal/mol, which was higher than its affinity with elongation factor EF-Tu (−8.12 kcal/mol), as shown in Table 7. These findings suggest that Funtumine, identified from *B. amplexicaulis*, can effectively interact with core microbial targets. Given these results, Funtumine was selected for further molecular dynamics simulation analysis.

**TABLE 7:** Binding energies, hydrogen and hydrophobic interactions between the top four identified molecules and the microbial drug target active site residues.

Ligand	Protein	Binding Energy (kcal/mol)	Hydrogen bond	Hydrophobic interactions
Funtumine	Penicillin-binding protein	−8.14	—	GLY:453, LYS:482, TYR:496, ASP:455, PRO:499, LYS:498, SER:479, ALA :485, GLU:481, LYS:474, LYS:475
	DNA Gyrase	−8.23	GLU:58, ASP81, GLY:85, LYS93, THR173	PHE:160, ASP:161, LEU:162, VAL:165, LEU:60, ASP:53, GLU:50, LYS:163
	Elongation factor (EF-Tu)	−8.12	GLN:67, VAL:500	TYR:505, GLU:420, MET:372, LYS:499, GLU:325, TRP:318, HIS:322, HIS:373, PRO:501, ASP:502, THR:411, PRO:503, TYR:69
	Sterol 14-alpha demethylase	−8.17	GLU:1221, LYS:1190	HIS:1238, THR:1170, ALA:1181, GLY:1177, GLU:1173, GLN:1174, ASP:1222, ILE:1223, HIS:1192, ARG:1154, GLY:1152
	Fungal 1,3-beta-glucan synthase	−8.15	TYR:140, SER:382	GLY:314, PHE:236, MET:509, LEU:380, HIS:381, PHE:384, LEU:383, LEU:129, PHE:241, TYR:126, THR:130, PHE:134
	Lanosterol14-alpha demethylase	−8.16	HIS:273, GLU:226	GLU:271, MET:272, ARG:274, THR:239, GLY:240, ARG:241, ASN:285, VAL:286, GLY:287
3-Hydroxycoumarin	Penicillin-binding protein	−3.68	GLU:481,	ALA:485, GLY:453, LYS:482, TYR:496, PRO:499, LYS:474, LEU:452
	DNA Gyrase	−4.14	GLU:201	ARG:42, THR:194, TRP:49, ARG:198, GLN:197
	Elongation factor (EF-Tu)	−4.80	LYS:9, GLU:271, HIS:273,	GLY:287, THR:239, VAL:286, ASN:285, MET:272, ARG:274, LYS:275, ARG:234
	Sterol 14-alpha demethylase	−6.46	TYR:69, MET:374,	PRO:503, THR:411, MET:372, HIS:373, GLY:407, VAL:510, TYR:505
	Fungal 1,3-beta-glucan synthase	−5.66	GLU:1173	TYR:696, PHE:1176, TYR:693, ALA:1293, PHE:1297, HIS:1294, ILE:1223, GLY:1177, LEU:1184, PHE:1180
	Lanosterol14-alpha demethylase	−4.96	HIS:381, SER:508	PHE:384, PHE:241, LEU:380, TYR:126, LEU:383, SER:382, THR:511, PRO:379, VAL:510, PHE:506, MET:509
N-Feruloyltyramine	Penicillin-binding protein	−3.51	GLU:481, LYS:498,	LYS:474, VAL:362, SER:479, LYS:475, LYS:482, PRO:499, GLY:453, LEU:452, ALA:485, TYR:496, ILE:454
	DNA Gyrase	−4.88	GLU:72, ARG:198	LYS:78, ARG:176, GLU:139, TYR:141, GLN:197, THR:194, GLU:201, ARG:198, LEU:205
	Elongation factor (EF-Tu)	−6.26	ASN:285, ARG:274, LYS:9	ILE:231, GLU:271, MET:272, PRO:10, HIS:273, VAL:286, GLY:287, ARG:274
	Sterol 14-alpha demethylase	−6.70	GLU:73, GLU:70, MET:372, TYR:69	ARG:414, TYR:408, SER:412, GLU:413, TYR:505, HIS:373, HIS:322, GLU:420, PRO:419, PRO:503, GLN:67, THR:411
	Fungal 1,3-beta-glucan synthase	-6.20	GLN:1174, ASP:1222, LYS:1190, GLU:1221	THR:1175, THR:1178, GLY:1177, GLU:1173, ALA:1181, HIS:1192, HIS:1238, LEU:1191, ILE:1223,
	Lanosterol14-alpha demethylase	−6.79	MET:313, THR:318, PHE:506, SER:508, TYR:126	PHE:236, GLY:314, GLN:316, GLY:315, HIS:317, PHE:244, LEU:380, PH:384, MET:509, VAL:510, PRO:238, THR:507, THR:511, HIS:381, SER:382, PRO:379, LEU:383
Methoxybrassenin A	Penicillin-binding protein	−3.79	TYR:496, LYS:482	PRO:499, LYS:498, TYR:501, LYS:474, GLU:481, LYS:475, ASP:455, ASN:450, ILE:454, ALA:485, GLY:453
	DNA Gyrase	-4.32		ARG:198, ILE:148, TYR:141, HIS:143, VAL:174, LYS:78, ARG:176, PHE:204, LEU:205, GLU:201
	Elongation factor (EF-Tu)	−5.35	LYS:9, HIS:273	VAL:249, ASN:285, MET:272, GLY:287, GLU:271, ARG:274,
	Sterol 14-alpha demethylase	−5.31		MET:374, VAL:510, TYR:505, PRO:503, PRO:501, HIS:373, VAL:500, MET:372, ASP:502, GLU:420, HIS:322, TYR:69, THR:411
	Fungal 1,3-beta-glucan synthase	−5.30	GLU:1155	GLY:1177, ALA:1153, HIS:1238, THR:1178, ARG:1154, PRO:1196, HIS:1195, HIS:1192, LYS:1190, ILE:1223, ALA:1181
	Lanosterol14-alpha demethylase	−5.06	TYR:140	LEU:383, SER:382, PRO:238, HIS:381, MET:509, PHE:241, PHE:384, LEU:380, LEU:129, THR:130, TYR:126

### Molecular dynamic (MD) simulation analysis

#### Root mean square deviation (RMSD) analysis

**FIGURE 9:**
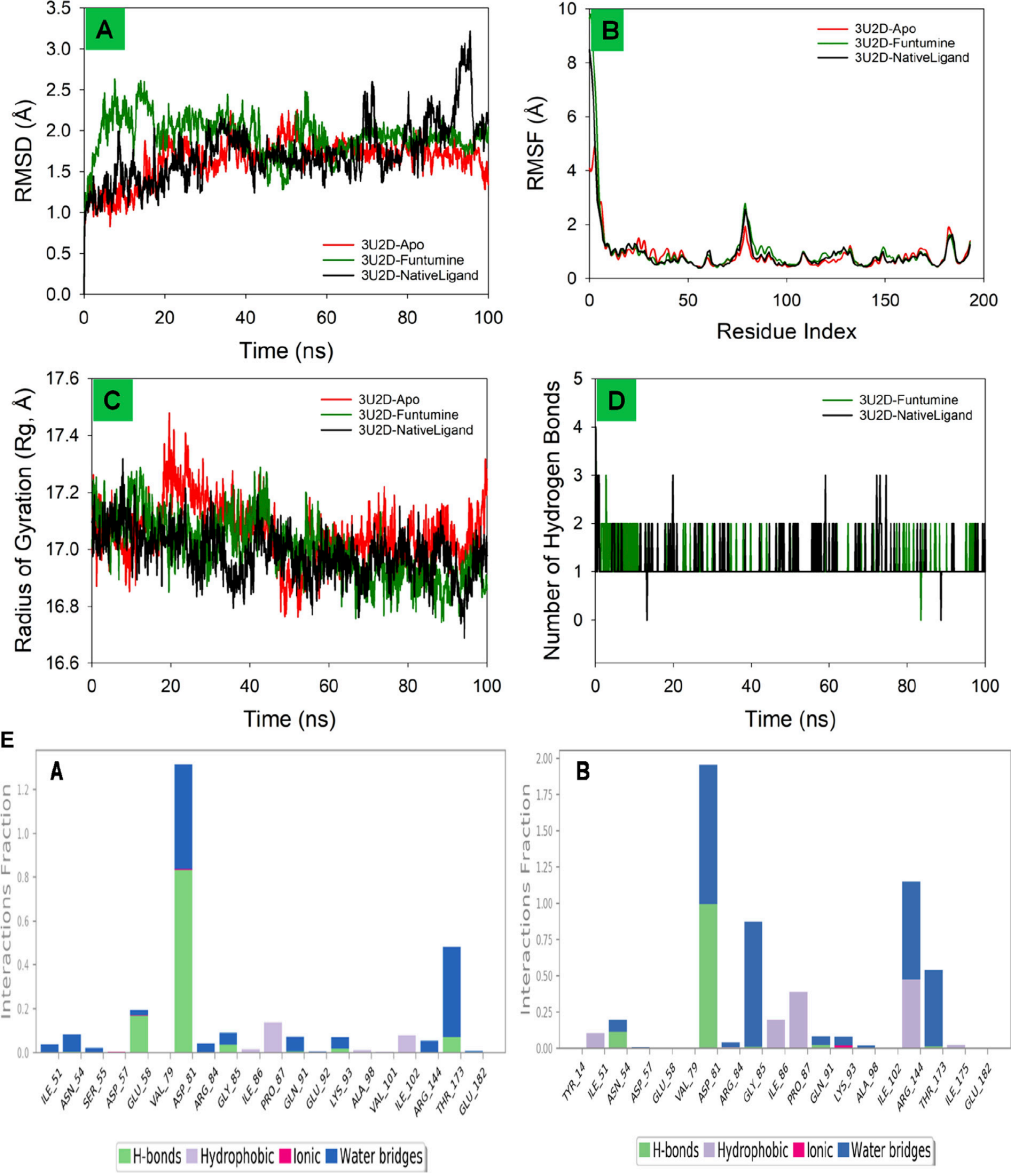
MD simulation analysis of 100 ns trajectories of **(A)** RMSD of the Cα backbone of DNA gyrase-Apo, DNA gyrase-Funtumine and DNA gyrase-Native ligand **(B)** RMSF of Cα backbone of DNA gyrase -Apo, DNA gyrase -Funtumine and DNA gyrase-Native ligand **(C)** Radius of Gyration of DNA gyrase-Apo, DNA gyrase-Funtumine and DNA gyrase-Native ligand **(D)**. Number of Hydrogen bonds of DNA gyrase -Apo, DNA gyrase-Funtumine and DNA gyrase -Native ligand. **(E)** The interactions involving protein-ligand contact were observed and are illustrated in the organized bar charts. Panels **(A,B)** display the interaction of Funtumine and the native ligand with the target protein.

The root mean square deviation (RMSD) is a fundamental metric in molecular dynamics (MD) simulations, offering insights into the structural stability and dynamic behavior of biomolecular systems over time. In this study, the RMSD profiles of bacterial DNA gyrase, the both in its Apo form and in complexes with Funtumine and its native ligand, were monitored over a 100 ns simulation. The Apo form (red line) shows a gradual increase in RMSD, stabilizing around 1.5 Å after 20 ns, indicating good structural stability without significant conformational changes, which aligns with typical behavior reported for stable protein structures in MD simulations (Hollingsworth and Dror, 2018). The 3U2D-Funtumine complex (green line) demonstrates higher fluctuations, with RMSD values ranging between 1.5 and 2.5 Å, suggesting moderate instability or conformational flexibility upon ligand binding. Such a range is considered acceptable in ligand-bound MD simulations but indicates that Funtumine induces notable structural perturbations (Hospital et al., 2012). The native ligand complex (black line) initially behaves comparably to the Apo form but shows increased deviations beyond 80 ns, peaking around 3.0 Å. This late-stage fluctuation might suggest either a ligand dissociation event or significant conformational rearrangement within the binding pocket, which warrants further analysis via binding free energy calculations or clustering of simulation frames. Overall, the simulation appears qualitatively sound, with initial equilibration within the expected 10–20 ns and RMSD values mostly within the 1–3 Å range, generally acceptable for protein-ligand systems (Lindorff‐Larsen et al., 2010). However, the pronounced late-stage instability in the native ligand complex should be critically evaluated in follow-up simulations or experimental validations to confirm its biological relevance (Figure 9A).

#### Root mean square fluctuation (RMSF) analysis

The Root Mean Square Fluctuation (RMSF) analysis of the protein 3U2D, both in its apo form (no ligand) and in complex with the ligands Funtumine and its native ligand, offers valuable insight into residue-level flexibility during molecular dynamics (MD) simulations. RMSF evaluates the average deviation of a residue over time from a reference position, indicating local flexibility and dynamic stability. As depicted in the plot, all three systems share a common trend in fluctuations across the residue index, with significant peaks at the N-terminal (∼residues 1–10) and modest peaks near residues ∼90 and ∼180. The highest RMSF values (∼10 Å) occur at the N-terminal, which is typically flexible and often solvent-exposed, consistent with other MD studies on similar proteins (Lindorff-Larsen et al., 2011). The central region of the protein (residues 30–150) exhibits lower fluctuations, suggesting a more stable and rigid core domain, crucial for functional integrity. Notably, the ligand-bound forms (Funtumine and native ligand) demonstrate a reduction in fluctuations compared to the apo form, particularly between residues 80–120. This indicates ligand-induced stabilization, potentially through key binding interactions that rigidify local secondary structures and reduce conformational entropy, which is a hallmark of stable protein-ligand complexes (Salo-Ahen et al., 2020). The similarity in fluctuation profiles between the Funtumine and NativeLigand-bound systems suggests that Funtumine may mimic the native ligand’s binding mode and influence on protein dynamics, making it a promising candidate for further structural and functional analysis. Overall, the RMSF profiles confirm a high-quality MD simulation with reasonable residue fluctuation patterns and clear conformational stabilization upon ligand binding. This is in agreement with established criteria for MD quality, such as stable RMSF values in the protein core and consistent trends across replicates (Hospital et al., 2012), reinforcing the reliability of these findings (Figure 9B).

#### Radius of gyration (Rg) ananlysis

The Radius of Gyration (Rg) is a key metric to evaluate the overall compactness and folding behavior of a protein during molecular dynamics (MD) simulations. In this analysis of the 3U2D protein, the Rg is monitored over a 100 ns simulation for three systems: the apo form (no ligand), and complexes with Funtumine and the native ligand. Across the simulation timeline, all three systems demonstrate Rg values fluctuating between ∼16.6 Å and ∼17.6 Å, with an overall downward trend indicating increasing compactness. The apo form (red) shows relatively higher and more fluctuating Rg values, especially in the early phase of the simulation (0–40 ns), suggesting a less compact and potentially more flexible or unstable structure. In contrast, the ligand-bound forms particularly the native ligand (black) and Funtumine (green) exhibit lower and more stabilized Rg profiles, indicative of enhanced structural compactness and stability. The average Rg values of the ligand-bound systems hover around 17.0 Å, compared to the apo form’s higher average, confirming ligand-induced folding and stabilization. This behavior is consistent with literature reporting that ligand binding often leads to a more compact protein conformation due to specific interactions that reduce the entropy and solvent exposure of flexible loops and termini (Liu et al., 2016). Furthermore, the similarity in Rg profiles of Funtumine and the native ligand suggests that Funtumine effectively mimics the native ligand’s binding dynamics and structural impact, potentially making it a functional analogue. The consistent and relatively narrow range of Rg fluctuations for the ligand-bound systems also indicates good simulation convergence and reliable folding dynamics, as recommended for robust MD studies (Amadei et al., 1993). Overall, the Rg data support the structural stability and compactness of 3U2D in ligand-bound states, reinforcing the notion that ligand binding is crucial for maintaining native-like conformations (Figure 9C).

#### Hydrogen bonds analysis

The number of hydrogen bonds formed between the 3U2D protein and its ligands Funtumine and the native ligand over a 100 ns molecular dynamics (MD) simulation provides crucial insights into the stability and binding affinity of the ligand-protein complexes. Hydrogen bonds are pivotal non-covalent interactions that significantly contribute to the specificity and stability of protein-ligand binding. From the graph, both ligand-bound systems show a consistent formation of 1–3 hydrogen bonds throughout the simulation, with occasional transient disruptions. The native ligand (black line) shows slightly more frequent fluctuations with intermittent spikes reaching 3 hydrogen bonds, while also occasionally dropping to zero. Funtumine (green line), on the other hand, demonstrates a more stable profile with fewer abrupt losses of hydrogen bonding, suggesting a steadier interaction throughout the simulation period. This consistency implies that Funtumine maintains a stable binding orientation within the active site, which is essential for biological function and inhibitory potential. The persistence of at least one hydrogen bond for the majority of the simulation timeframe in both systems indicates a reasonably stable binding, aligning with established molecular dynamics principles where sustained hydrogen bonding is correlated with high ligand affinity and reduced dissociation rates (McCammon and Harvey, 1988). Additionally, the hydrogen bond occupancy over time is within the range observed in successful drug-like interactions, further affirming the structural reliability of the simulation (Durrant and McCammon, 2011). Interestingly, although the native ligand shows momentary superiority in bond count, the overall steadiness of Funtumine’s hydrogen bonding may indicate favorable thermodynamic stability and pose it as a viable alternative for drug development. These results, combined with RMSF and Rg data, build a coherent picture of ligand-induced stability in the DNA gyrase protein, with hydrogen bonding serving as a critical determinant of interaction quality and persistence (Figure 9D). Compounds Funtumine compared to Native ligand demonstrated significant hydrogen bonding and other interactions with the target protein, as illustrated in (Figure 9E), highlighting their notable binding potential.

#### Solvent accessible surface area (SASA) analysis

**FIGURE 10:**
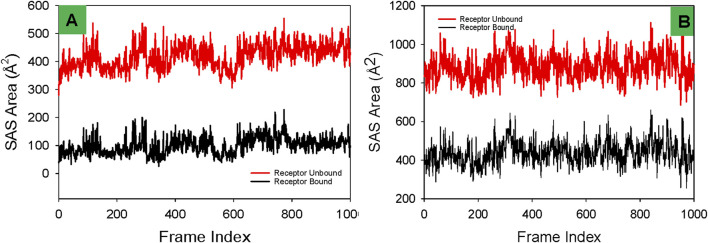
Comparative Solvent Accessible Surface Area (SASA) profiles of receptor proteins in ligand-bound and unbound states with **(A)** Funtumine and **(B)** Native Ligand across DNA gyrase target protein. Each plot illustrates the temporal evolution of SASA (in Å^2^) over a 1,000-frame molecular dynamics simulation for both the ligand-unbound (red) and ligand-bound (black) states of the receptor.

The Solvent Accessible Surface Area (SASA) profiles depicted for the 3U2D protein bound to NativeLigand and Funtumine provide a valuable understanding of the structural behavior and binding dynamics during molecular dynamics (MD) simulations. SASA reflects the degree of exposure of a protein’s surface to the solvent and changes in SASA values can indicate ligand-induced conformational adjustments. In the left panel (3U2D-NativeLigand), the receptor-bound state (black line) displays consistent and relatively lower SASA values (∼100–250 Å^2^), indicating substantial burial of the ligand within the protein and thus a well-stabilized complex. The receptor-unbound form (red line), in contrast, shows higher SASA values (∼350–500 Å^2^), as expected due to increased surface exposure without ligand stabilization. The tight clustering and minimal fluctuations in the receptor-bound state suggest a stable, compact conformation, reinforcing the idea that the native ligand is strongly integrated into the protein’s binding site. Comparatively, in the right panel (3U2D-Funtumine), the receptor-bound SASA values are significantly elevated (∼300–600 Å^2^), and receptor-unbound values are even higher (∼850–1,100 Å^2^). The higher SASA values in both bound and unbound states suggest that Funtumine binding induces or sustains a more solvent-exposed and flexible structure than the native ligand. This could point to a less snug fit or weaker interaction with the binding site, possibly destabilizing the protein to a degree. The larger amplitude of SASA fluctuations in the Funtumine-bound state, relative to the native ligand complex, further indicates that the protein structure experiences more dynamic movements and less structural rigidity upon Funtumine binding. These observations imply that while Funtumine does interact with the protein, its effect on the global folding and compactness is less favorable compared to the native ligand. In MD simulations, such distinctions in SASA profiles are vital markers of ligand binding quality and stability, as robust and low SASA values in the bound form generally correlate with energetically favorable interactions and reduced solvent interference. Studies such as Durrant and McCammon (2011), Wang and Hou (2012) support the significance of SASA in understanding binding thermodynamics and structural coherence in simulations. Overall, the SASA data affirm that the native ligand ensures better conformational stability and stronger integration into the 3U2D receptor compared to Funtumine, highlighting its superior binding efficacy (Figure 10).

#### Dynamical cross-correlation map (DCCM) analysis

**FIGURE 11:**
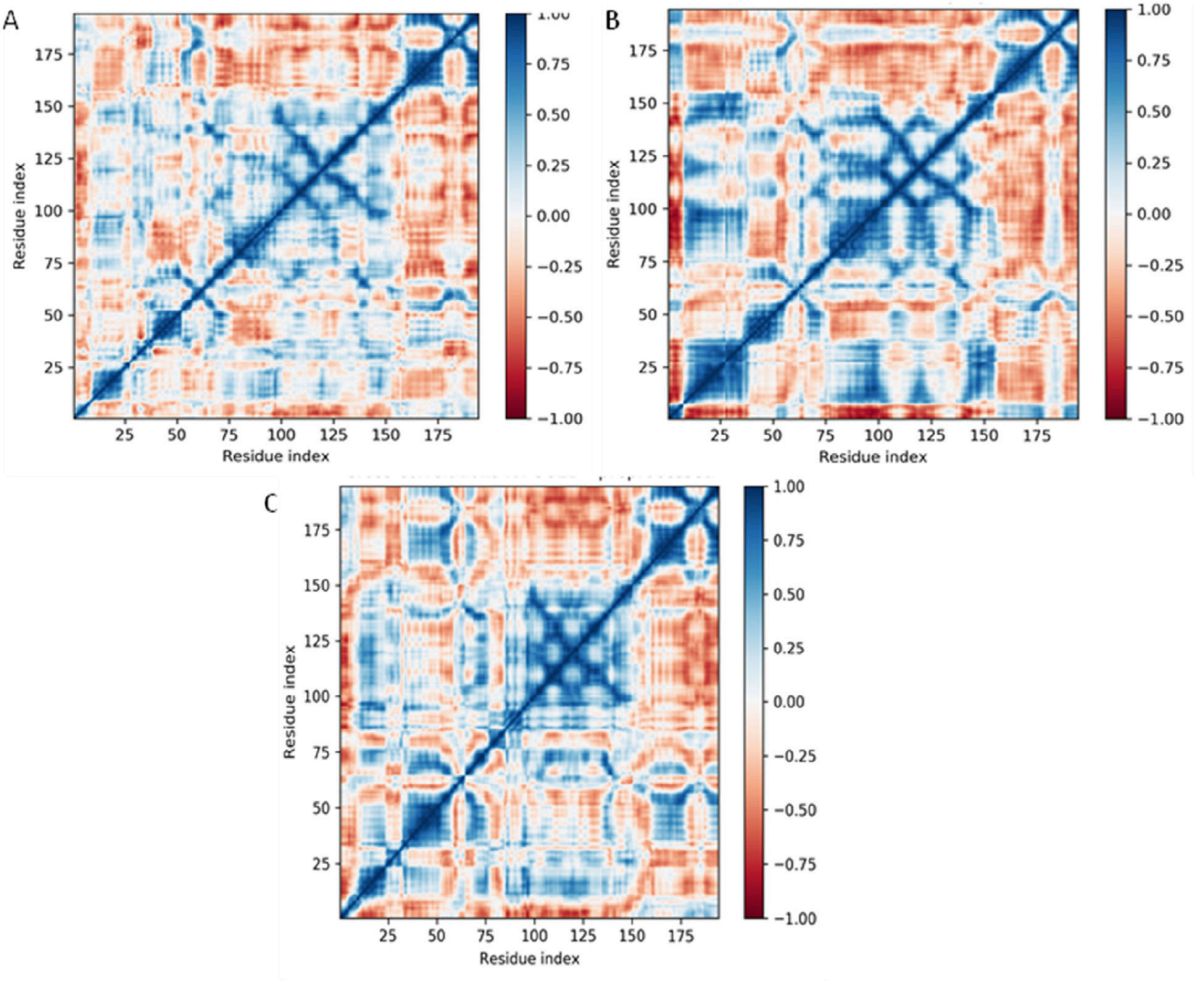
Dynamical Cross-Correlation Maps (DCCMs) of Cα atoms for DNA gyrase protein under different ligand-binding conditions derived from 100 ns molecular dynamics simulations. **(A)** DCCM for DNA gyrase-Apo (ligand-free form), **(B)** DCCM for DNA gyrase in complex with Funtumine, and **(C)** DCCM for DNA gyrase in complex with the native ligand. Each matrix element represents the cross-correlation coefficient of atomic displacements between residue pairs. Positive correlations (blue) indicate residues moving in the same direction, while negative correlations (red) indicate anti-correlated motions.

The Dynamical Cross-Correlation Map (DCCM) analysis provides insights into the internal dynamics and correlated motions of residues within a protein over the course of a molecular dynamics (MD) simulation. In this figure, the DCCMs of the DNA gyrase protein in its Apo form (no ligand) (Figure 11A), and when bound to Funtumine or the native ligand, are visualized. The DCCM captures correlations between atomic fluctuations of residue pairs: values approaching +1 (blue) indicate strong correlated motions (same direction), while values near −1 (red) indicate strong anti-correlated motions (opposite direction). In the Apo form, the correlation map shows less organized blue and red zones, particularly in mid to high residue ranges (residues 50–180), indicating a lack of coherent domain motion and possibly more disordered internal dynamics. In contrast, the DCCM of the Funtumine-bound DNA gyrase protein ((Figure 11B), shows more pronounced and structured blue regions, especially around residue ranges 25–100 and 130–175, suggesting enhanced cooperative movements between distant regions of the protein. These correlations imply that ligand binding helps stabilize specific intra-domain motions that may be critical for functional conformations. The NativeLigand-bound (Figure 11C), form exhibits the most extensive and sharply defined cross-correlation regions among the three, indicating that the native ligand induces the most functionally relevant dynamic coupling within the protein. Regions such as 25–50 and 100–150 in this complex show highly coordinated motion patterns, reflecting the ligand’s capability to enhance intramolecular communication, possibly through allosteric networks. Comparing these observations with literature, correlated motions are often linked to allosteric regulation and protein function, and ligand-induced enhancement of such correlations has been associated with increased protein stability and functional efficiency (Bahar et al., 2010; Bakan et al., 2011). Therefore, the native ligand exhibits the most optimal binding, enhancing both structural and dynamic features of the 3U2D protein. However, Funtumine also promotes substantial dynamic correlation, positioning it as a promising alternative ligand. The Apo form, lacking these stabilizing influences, displays weaker correlation patterns, highlighting the critical role of ligand interactions in modulating the dynamic architecture of the protein. In summary, DCCM analysis clearly illustrates that both Funtumine and the native ligand significantly enhance internal motion coordination in 3U2D, with the native ligand showing superior modulation of long-range correlations, while Funtumine presents a compelling, functionally relevant dynamic profile.

#### Principal component analysis (PCA) analysis

**FIGURE 12:**
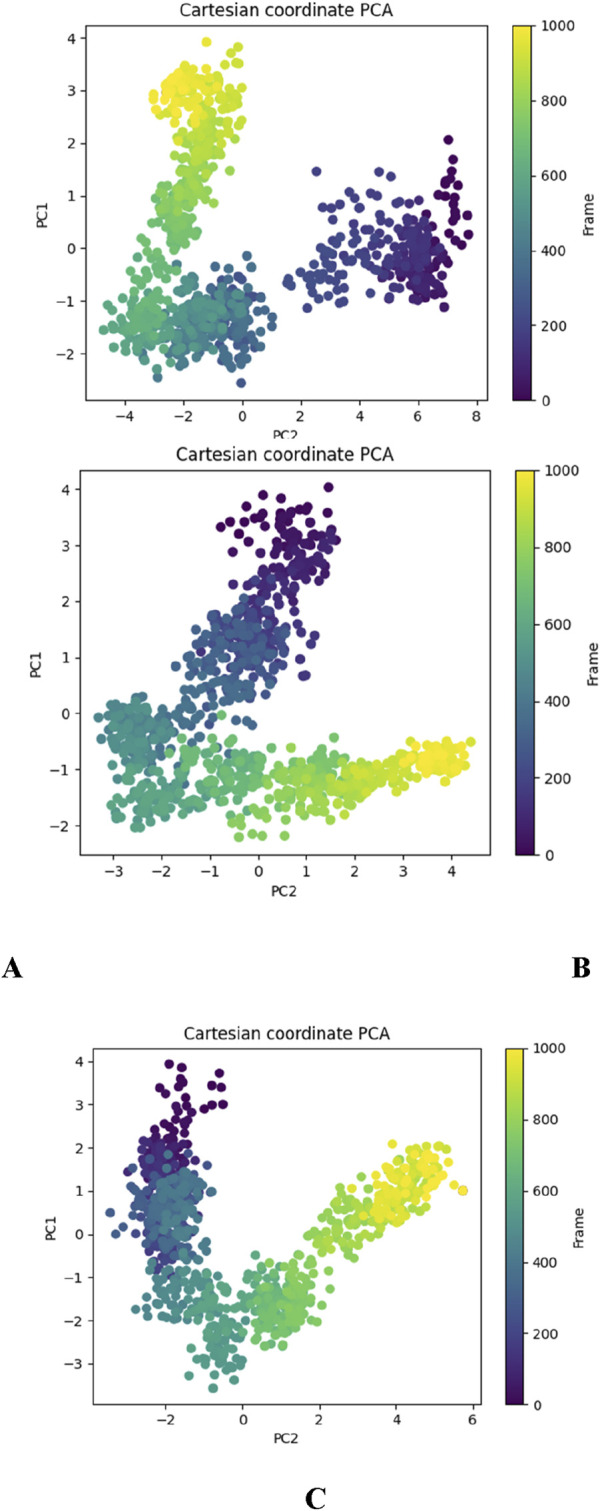
Principal Component Analysis (PCA) plots depicting the essential dynamics of the 3U2D protein backbone atoms over the course of 1000-frame molecular dynamics simulations for three systems: **(A)** DNA gyrase-Apo, **(B)** DNA gyrase-Funtumine, and **(C)** DNA gyrase-Native Ligand.

The Principal Component Analysis (PCA) plots derived from the molecular dynamics (MD) simulations of the DNA gyrase protein complexed with Apo, Funtumine, and NativeLigand states provide critical insights into the conformational landscape and dynamic motions of the protein throughout the simulation trajectory. PCA reduces the high-dimensional atomic motion data into principal components (PCs), primarily PC1 and PC2 here, which represent the most significant collective motions. In the DNA gyrase protein-Apo (top-left), the PCA plot reveals a broader and more dispersed conformational space, indicating higher flexibility and structural variability in the absence of a ligand. The trajectory shows a transition across distinct regions, suggesting the protein samples multiple conformational states, reflecting its intrinsic dynamic nature in the unbound state (Figure 12A). In contrast, the DNA gyrase -Funtumine complex (top-right) demonstrates a relatively more constrained but still diversified motion pattern. The transition pathway is more linear and directional, suggesting some stabilization by the ligand, although the system still explores alternative conformations (Figure 12B). The spread across PC2 and the more linear distribution imply that Funtumine restricts the motion to specific modes but does not fully stabilize the protein as rigidly as the native ligand. The DNA gyrase-NativeLigand (bottom center) complex, however, shows a compact and well-defined trajectory with minimal overlap across states, forming a curved trajectory with two major clusters (Figure 12C). This signifies a more confined and consistent conformational sampling, indicative of enhanced stability and limited fluctuation induced by tight ligand binding. The smooth transition from early to later frames (purple to yellow) in a continuous manner demonstrates that the system equilibrates and maintains a specific dynamic behavior. These findings confirm that the native ligand imposes stronger structural restrictions and stabilizes the protein conformation more effectively than Funtumine, while the Apo form retains maximum flexibility. PCA, thus, not only corroborates the ligand-induced conformational effects but also provides a robust measure of the structural integrity and quality of the MD simulation.

#### Molecular mechanics generalized born surface area (MM/GBSA) calculations

Utilizing the MD simulation trajectory, the binding free energy along with other contributing energy in form of MM/GBSA were determined for 3U2D bound to Funtumine (Table 8). The presented table summarizes the Molecular Mechanics Generalized Born Surface Area (MM/GBSA) binding free energy components for the 3U2D protein bound to two different ligands, Funtumine and Native Ligand. MM/GBSA is a widely accepted post-processing technique for estimating the binding free energy of biomolecular complexes from molecular dynamics simulations, offering insights into the energetic contributions that govern ligand affinity and stability within a binding pocket. The total binding free energy (ΔGbind) for the 3U2D-Funtumine complex is slightly more favorable at −58.45 kcal/mol compared to −56.61 kcal/mol for 3U2D-NativeLigand, suggesting that while both ligands form stable complexes, Funtumine displays marginally higher affinity for the receptor. Decomposing the energies reveals nuanced differences in interaction profiles. The lipophilic (ΔGbindLipo) contribution is substantially more favorable in the 3U2D-Funtumine complex (−26.22 kcal/mol) than in 3U2D-NativeLigand (−15.23 kcal/mol), indicating stronger hydrophobic interactions for Funtumine. Conversely, van der Waals (ΔG_bind_vdW) interactions are notably more favorable for NativeLigand (−23.21 kcal/mol) than Funtumine (−14.45 kcal/mol), implying a tighter steric complementarity between NativeLigand and the binding pocket. Electrostatic (Coulombic) interactions are repulsive for both, with positive values (16.65 kcal/mol for Funtumine and 11.54 kcal/mol for NativeLigand), but slightly less destabilizing in the NativeLigand complex. Hydrogen bonding contributions (ΔG_bind_Hbond) are modest yet more favorable for Funtumine (−4.01 kcal/mol vs. −2.99 kcal/mol), reflecting more or stronger hydrogen bonds in that complex. The solvation energy (ΔG_bind_SolvGB), typically opposing binding due to desolvation penalties, is lower for NativeLigand (38.12 kcal/mol) than for Funtumine (48.23 kcal/mol), suggesting better solvation balance upon binding. Covalent (ΔG_bind_Covalent) and packing (ΔG_bind_Packing) terms remain comparable, with packing slightly favoring NativeLigand (−7.28 kcal/mol). Overall, while Funtumine achieves better lipophilic and hydrogen bonding interactions, NativeLigand compensates with superior van der Waals and solvation energy terms, indicating distinct but competitive binding profiles, consistent with established MM/GBSA applications in ligand affinity prediction.

**TABLE 8:** Binding free energy components for the DNA Gyrase bound to Funtumine and Native Ligand calculated by MM/GBSA analysis.

Energies (kcal/mol)	DNA Gyrase + Funtumine	DNA Gyrase + Native Ligand
ΔG_bind_	−58.45	−56.61
ΔG_bind_Lipo	−26.22	−15.23
ΔG_bind_vdW	−14.45	−23.21
ΔG_bind_Coulomb	16.65	11.54
ΔG_bind_H_bond_	−4.01	−2.99
ΔG_bind_SolvGB	48.23	38.12
ΔG_bind_Covalent	2.09	2.22
ΔG_bind_Packing	−6.99	−7.28

## Discussion

Natural products are essential to chemical biology and drug discovery; many modern medications either mimic compounds found in nature or have structures that are wholly or partially derived from natural patterns, as noted by (Kamaraj et al., 2012). The ongoing quest for new antibacterial compounds is a response to the growing problem of antibiotic-resistant microbes. Plant-derived glycosides, alkaloids, terpenes, flavonoids polyphenols, and tannins have been identified for their antibacterial properties; however, phenolic acids, flavonoids, and tannins have been primarily associated with antimicrobial activity (Daglia et al., 2007; Li et al., 2012). Some of these compounds have demonstrated synergistic effects when used in conjunction with existing antimicrobial drugs, as reported by Ncube et al., 2008. The occurrence of several secondary metabolites, including phenolics, alkaloids, flavonoids, glycosides, saponins, and tannins, was shown by phytochemical analysis of *B. amplexicaulis* extracts.

Numerous parameters affect the effectiveness of phytochemical extraction, such as the phytochemical type, the sample particle dimensions, extraction technique, extraction duration, temperature, pH, the ratio of solute to solvent, and solvent polarity (Do et al., 2014). The careful selection of solvent systems is necessary for the effective extraction of phytochemicals, including polyphenols, from the samples. In solvent extraction, the selection of an appropriate solvent is a critical factor, influenced by parameters such as selectivity, solubility, and safety. Based on the principles of similarity and intermiscibility, solvents with polarity values comparable to those of the target solute exhibit enhanced extraction efficiency (Lee et al., 2024). Frequently employed solvents in extraction processes include ethanol, methanol, water, and acetone. Methanol, a highly polar solvent with significant solubility, is widely utilized for the extraction of various polar compounds. Despite its high extraction efficiency, its application requires caution due to its toxic nature. It is commonly employed for the extraction of phenolic compounds, lipids, fatty acids, anthocyanins, terpenoids, lignans, polysaccharides, proteins, and amino acids (Carrera et al., 2012; Spigno et al., 2007). Similarly, a medium-polar solvent (Ethyl acetate in the present study), is characterized by its high volatility, facilitating easy removal post-extraction. It is particularly suitable for extracting medium-polar compounds, with notable efficiency in isolating phenolic compounds and flavonoids (Kumar et al., 2023).

Owing to their numerous biological properties, flavonoids and other polyphenols obtained from plants should be assessed for their presence in various plant sections that have been extracted using a range of organic solvents. Owing to their numerous biological properties, flavonoids and other polyphenols obtained from plants should be assessed for their presence in various plant parts (Azadi Gonbad et al., 2015). In this regard, four different solvents with increasing polarity were used. Ethyl acetate showed the highest concentration of most of the phytochemicals, followed by methanolic extracts. Therefore, both ethyl acetate and methanolic extracts were further analyzed for antimicrobial activity.

**FIGURE 13:**
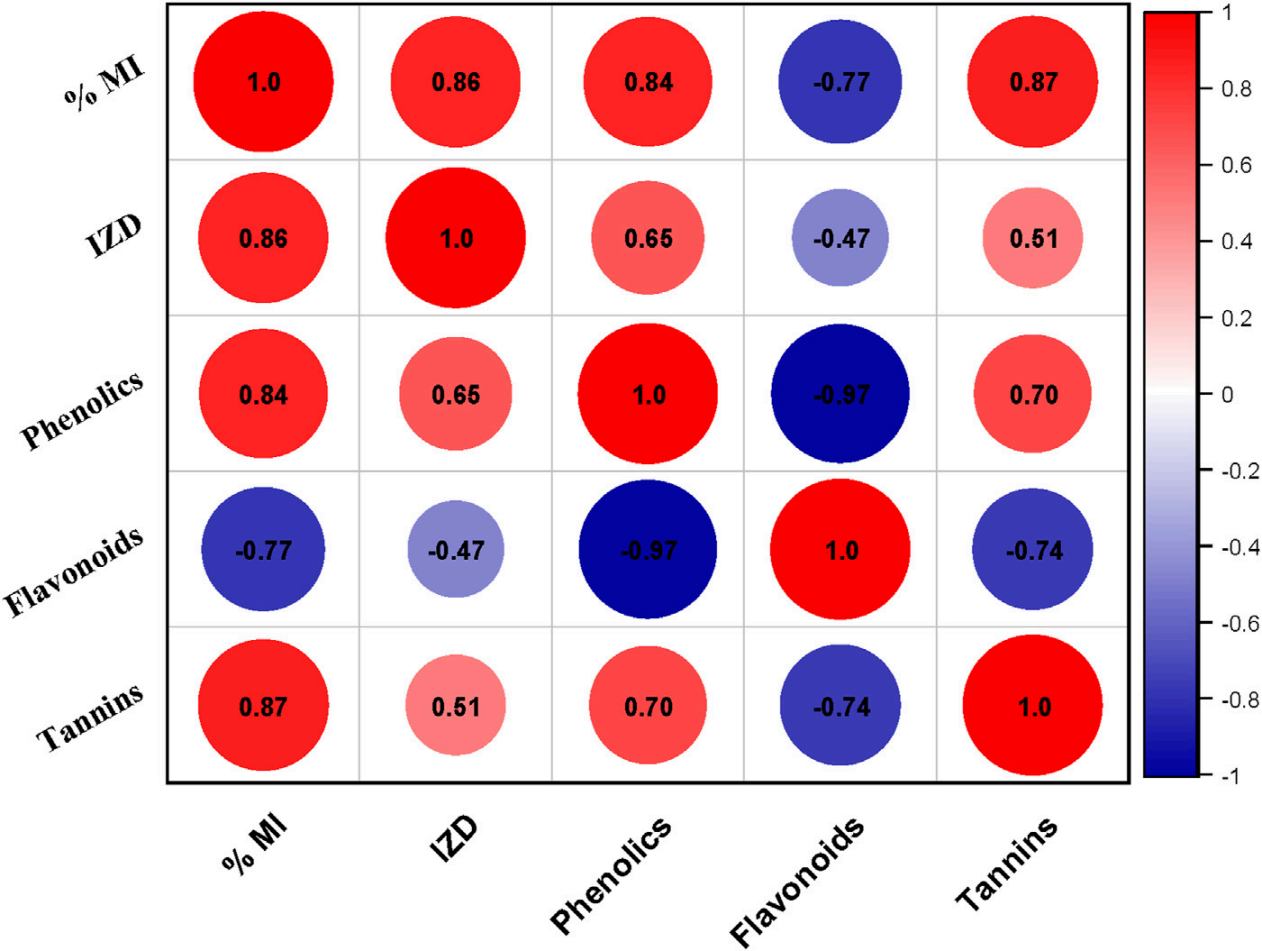
Correlation plot between different phytochemicals (Phenolics, Flavonoids, Tannins) inhibition zone diameter (IZD) and % Mycelial inhibition (% MI) of different bacterial and fungal strains respectively.

The potent antibacterial effects observed in the ethyl acetate extract are attributed to the presence of phenolic compounds, flavonoids, and tannins, as documented by previous studies (Mujeeb et al., 2014). A correlation plot (Figure 13) depicts the relation between phenolics, flavonoids, tannins, and inhibition towards different bacterial and fungal strains. Phenolics characterized by their aromatic structures and multiple hydroxyl groups, neutralize free radicals and reactive oxygen species by donating electrons or hydrogen atoms (Vuolo et al., 2019). These compounds inhibit efflux pumps, cell wall biosynthesis, and key bacterial enzymes like urease and dihydrofolate reductase. Flavonoids and non-flavonoids are prominent phenolic compounds (Barbieri et al., 2017). Cell wall production is disrupted by tannins when they form irreversible compounds with proteins rich in proline. Saponins cause cells to leak proteins and enzymes, whereas terpenoids break down microbial cell walls via weakening membranes (Mujeeb et al., 2014).

Flavonoids, synthesized by plants in response to infections, complex with extracellular proteins and bacterial cell walls leading to cell wall disruption (Hernandez et al., 2000). Steroids, specifically affecting membrane lipids, cause leakage from liposomes (Epand et al., 2007). Saponins, which are natural cleansing agents, also demonstrate effectiveness as antimicrobial substances (Aqil et al., 2010). Triterpenoid compounds have also been reported to possess antifungal activity as they can affect the permeability of the cell membrane which could lead to cell membrane lysis (Muchtaromah et al., 2017).

By employing the HR-LC/MS method, the phytochemical components within the ethyl acetate extract of both belowground and aboveground portions of *B. amplexicaulis* were scrutinized and subsequently characterized. Various bioactive compounds in medicinal plants have been identified, including 4H-Pyran-4-one, 2, 3-dihydro-3, 5-dihydroxy-6-methyl-, with anti-inflammatory and antioxidant properties in *Barleria prionitis* (Linn.) rhizome. 1-Dodecanol exhibits high antibacterial activity against *S. aureus* (Togashi et al., 2007). Butylated hydroxytoluene (BHT), an antioxidant, inhibits Gram-positive bacteria growth more effectively than gram-negative bacteria in the Enterobacteriaceae family. Derivatives of cinnamic acid possess antibacterial, antifungal, and antiviral properties (Sova, 2012). 2(4H)-Benzofuranone, 5,6,7,7a-tetrahydro-4,4,7a-trimethyl, identified in *Azadirachta indica*, exhibits analgesic, antidiabetic, antibacterial, and antifungal properties (Moorthy et al., 2011). According to Hamdan et al. (2011) and Navarro-Garcıa et al. (2011), stigmasterol has antibacterial and high antioxidant properties that work against mycobacteria that are resistant to many drugs.

As depicted in the study, most extracts of *B. amplexicaulis* possess better inhibition potential against gram-positive bacteria. A similar trend has also been reported by several other workers (Togashi et al., 2007; Biswas et al., 2013; Bobis et al., 2015; VARGhESE et al., 2013). Interestingly, because of their more intricate lipopolysaccharide-based cell membrane structure, gram-negative bacteria are more resistant to the extract than gram-positive bacteria. The lipopolysaccharide layer Gram-negative bacteria prevent hydrophobic substances like the extract from diffusing across their cell membranes. Furthermore, bacterial cell resistance to antibacterial chemicals is influenced by the rate of solubility and concentration of antimicrobial agents in the lipid moiety of cell membranes as well as the hydrophobicity of the membrane surface (Fathi et al., 2024).

To gain a deeper comprehension of the molecular basis of the biological activity of natural products, *in silico* investigations have proven to be an excellent means of predicting the theoretical interactions between ligands and targets (Saifi et al., 2024; Olğaç et al., 2017) Additionally, they offer more information about the potential mode of action and binding mode of active compounds against different microbiological drug targets (Khameneh et al., 2019) to correlate the findings of the experimental antimicrobial activity and to gain a deeper understanding of the antimicrobial activity of the compounds under study.

Based on the virtual screening, Funtumine was identifiied through HR/LC-MS analysis. Several methods by which an antibacterial drug might block cell wall formation; for years, these mechanisms have been recognized as critical antibacterial targets. In the present study, we have selected various antibacterial and antifungal target proteins. The selected antibacterial proteins are penicillin-binding protein (PBP), DNA Gyrase, and elongation factor-Tu (Eu-Tu). While selected antifungal proteins are Sterol 14-alpha demethylase, Fungal Lanosterol 14-alpha demethylase, and 1,3-beta-glucan synthase.

Membrane-associated proteins called penicillin-binding proteins (PBPs) are essential for the formation of the cell wall (Macheboeuf et al., 2005; Miyachiro et al., 2019). Amino-acylated tRNAs are mostly transported to the ribosome by EF-Tu. Antibiotics have targeted EF-Tu as a therapeutic target since the 1970s (Harvey et al., 2019). An essential bacterial enzyme called DNA gyrase helps double-stranded closed-circular DNA to undergo its ATP-dependent negative super-coiling. DNA gyrase can be targeted for the development of broad-spectrum antibiotics due to the high sequence and structural similarity across bacterial infections. The inhibition of the enzyme DNA gyrase affects the functional genomics of the bacteria as well as the overall expression of genes (Salman et al., 2023). Members of the cytochrome P450 family like Sterol 14α-demethylase (CYP51) and Lanosterol 14-alpha-demethylase (Erg11) are key enzymes in the ergosterol biosynthesis pathway.

The stability of protein-ligand complexes and their intermolecular interactions can be more effectively understood through MD simulation in drug development. Moreover, when a complex system is placed in a synthetic environment, it can predict the changes that will occur. MD simulation was utilized to evaluate with a time increment of 100 ns. The MD simulation trajectory evaluated the protein and ligand’s root mean square deviation (RMSD), the root mean square fluctuation (RMSF), the radius of gyration, the solvent accessible surface area (SASA), and the analysis of hydrogen bonds.

The molecular dynamics (MD) and post-MD analyses of the 3U2D protein, in its apo form and complexed with Funtumine compound and the native ligand, reveal significant insights into the stability and binding behavior of these systems. RMSD and RMSF analyses collectively indicate that ligand binding reduces overall structural fluctuations and stabilizes specific protein regions, particularly in the core domains. While the apo form remains relatively stable, both ligand-bound systems show reduced residue-level flexibility, with Funtumine demonstrating comparable effects to the native ligand in key functional regions. Radius of Gyration (Rg) results further support these findings, showing that ligand binding induces a more compact protein structure, with Funtumine maintaining a similar level of structural integrity as the native ligand.

Hydrogen bond and Solvent Accessible Surface Area (SASA) analyses reinforce the binding quality of the ligands. Funtumine forms slightly fewer but more consistent hydrogen bonds than the native ligand, suggesting steadier binding over the simulation period. However, higher SASA values for the Funtumine-bound complex indicate a more solvent-exposed binding conformation compared to the native ligand, implying a looser fit within the active site. Dynamical Cross-Correlation Map (DCCM) and Principal Component Analysis (PCA) show that both ligands enhance residue-residue motion coordination, but the native ligand promotes stronger and more structured dynamic coupling, suggesting tighter conformational control and possibly greater functional relevance.

Post-MD MM/GBSA analysis offers a detailed energetic profile of binding interactions. Funtumine displays slightly more favorable total binding free energy than the native ligand, driven by stronger lipophilic and hydrogen bonding contributions. Conversely, the native ligand benefits from more favorable van der Waals and solvation energy terms. These complementary interaction profiles highlight Funtumine as a promising alternative, with a binding affinity comparable to the native ligand, though further optimization may be required to enhance its structural integration and reduce solvent exposure. Collectively, the results underscore Funtumine’s potential as a lead compound with competitive stability and binding characteristics, warranting further investigation.

### Future prospects

In the present study, the antibacterial and antifungal activities of *B. amplexicaulis* were evaluated through *in vitro* experimental assays. The results obtained provide valuable preliminary insights into the antimicrobial potential of this species. However, to substantiate these findings and establish their clinical relevance, it is imperative to conduct *in vivo* studies, which would allow for a more comprehensive understanding of the efficacy and therapeutic applicability of *B. amplexicaulis* under physiological conditions. Furthermore, the antimicrobial activity of *B. amplexicaulis* should be assessed against a broader spectrum of bacterial and fungal strains to determine its efficacy across diverse microbial taxa. Such an approach would ensure a more robust validation of the findings and contribute to the broader field of natural antimicrobial research.

## Conclusion

In recent decades, global interest in investigating medicinal plants has surged due to their antibacterial and antioxidant properties, low toxicity, and potential cost-effectiveness as alternatives to expensive synthetic drugs. This increased attention is a response to the pressing global issue of rising microbial resistance to antibiotics. In light of this, our study aimed to explore the specific antimicrobial properties of different parts of *B. amplexicaulis* and assess how varying solvent polarities influenced its inhibitory effects. Through LC-MS analysis, *B. amplexicaulis* was found to contain bioactive compounds, the majority of which have therapeutic potential. Our research demonstrates the significant antimicrobial activity of *B. amplexicaulis* against various bacterial and fungal strains. Notably, belowground parts displayed higher antimicrobial potential in comparison to aboveground counterparts. Ethyl acetate extracts exhibited superior activity compared to methanolic extracts, and this activity increased with higher extract concentrations.

Additionally, our *in silico* molecular binding interaction analysis revealed that Funtumine exhibits specificity towards DNA gyrase binding sites. The compound shows promise as a potent antimicrobial agent. Investigating these interactions further may provide important information on the processes by which prospective antimicrobial medications target resistant strains of bacteria and fungi.

## Data Availability

The original contributions presented in the study are included in the article/Supplementary Material, further inquiries can be directed to the corresponding author.
