# Supplementary figures and images for: Metabolic profiling and antimicrobial activity of Bistorta amplexicaulis D. don by in-vitro implicated through computational studies
Source: Front Pharmacol. 2025 Jul 1;16:1575727. doi: 10.3389/fphar.2025.1575727 (PMC12259670; doi:10.3389/fphar.2025.1575727)

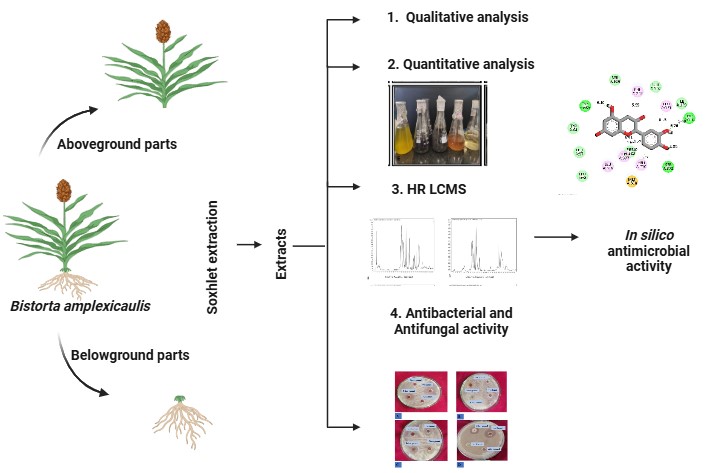

Supplement: Supplementary file 3 [file Supplementaryfile1.jpeg]
